# Nanobased Platforms for Diagnosis and Treatment of COVID-19: From Benchtop to Bedside

**DOI:** 10.1021/acsbiomaterials.1c00318

**Published:** 2021-05-12

**Authors:** Elham Bidram, Yasaman Esmaeili, Abbas Amini, Rossella Sartorius, Franklin R. Tay, Laleh Shariati, Pooyan Makvandi

**Affiliations:** ‡ Biosensor Research Center, School of Advanced Technologies in Medicine, Isfahan University of Medical Sciences, Hezarjerib Avenue, Isfahan 8174673461, Iran; § Centre for Infrastructure Engineering, Western Sydney University, Locked Bag 1797, Penrith 2751, New South Wales, Australia; ⊥ Department of Mechanical Engineering, Australian College of Kuwait, Al Aqsa Mosque Street, Mishref, Safat 13015, Kuwait; || Institute of Biochemistry and Cell Biology (IBBC), National Research Council (CNR), Via Pietro Castellino 111, Naples 80131, Italy; # The Graduate School, Augusta University, 1120 15th Street, Augusta, Georgia 30912, United States; □ Applied Physiology Research Center, Isfahan Cardiovascular Research Institute, Isfahan University of Medical Sciences, Hezarjerib Avenue, Isfahan 8174673461, Iran; ○ Department of Biomaterials, Nanotechnology and Tissue Engineering, School of Advanced Technologies in Medicine, Isfahan University of Medical Sciences, Hezarjerib Avenue, Isfahan 8174673461, Iran; △ Centre for Materials Interfaces, Istituto Italiano di Tecnologia, viale Rinaldo Piaggio 34, Pontedera 56025, Pisa, Italy

**Keywords:** COVID-19, coronavirus, nanobiosensor, nanobased vaccine, SARS-CoV-2

## Abstract

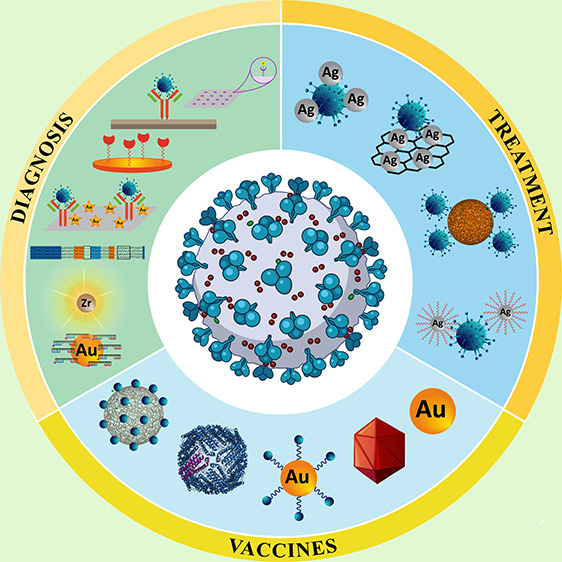

Human respiratory viral infections are the leading cause of morbidity and mortality around the world. Among the various respiratory viruses, coronaviruses (e.g., SARS-CoV-2) have created the greatest challenge and most frightening health threat worldwide. Human coronaviruses typically infect the upper respiratory tract, causing illnesses that range from common cold-like symptoms to severe acute respiratory infections. Several promising vaccine formulations have become available since the beginning of 2021. Nevertheless, achievement of herd immunity is still far from being realized. Social distancing remains the only effective measure against SARS-CoV-2 infection. Nanobiotechnology enables the design of nanobiosensors. These nanomedical diagnostic devices have opened new vistas for early detection of viral infections. The present review outlines recent research on the effectiveness of nanoplatforms as diagnostic and antiviral tools against coronaviruses. The biological properties of coronavirus and infected host organs are discussed. The challenges and limitations encountered in combating SARS-CoV-2 are highlighted. Potential nanodevices such as nanosensors, nanobased vaccines, and smart nanomedicines are subsequently presented for combating current and future mutated versions of coronaviruses.

## 1 Introduction

Coronavirus pandemics have emerged rapidly in the 21st century, with catastrophic consequences.^1,2^ The first severe acute respiratory syndrome coronavirus (SARS-CoV) pandemic, SARS-CoV-1, occurred in southern China in late 2002 and infected more than 8000 people with ∼10% mortality globally.^3^ This was followed by the emergence of the Middle East respiratory syndrome coronavirus (MERS-CoV) in 2012 that infected about 2494 people with a mortality rate of 34.4%.^4,5^ In late 2019, the new SARS-CoV-2 pandemic, often referred to as coronavirus disease 2019 (COVID-19), emerged in Wuhan, China, and spread quickly to all countries around the world.^6^ As of February 26, 2021, the World Health Organization (WHO) reported a total of 112 million confirmed cases with 2.5 million deaths.^7^ Over the last 12 months or so, considerable efforts have been made to rapidly develop COVID-19 vaccines to protect and mitigate the effects of this deadly disease on the human population. However, with more than 2.5 million deaths to date, there is an urgent need for fast and reliable diagnostic and therapeutic approaches against SARS-CoV-2 infections. Many aspects of the currently available vaccine formulations remain to be clarified, including the safety of administration on the pediatric population and their effectiveness against emerging viral strains.

Nanotechnology has opened up new horizons in many different aspects of medical science, such as targeted gene delivery, targeted drug delivery, biosensor platforms, imaging, and diagnosis.^8,9^ Nanomaterials have been developed to combat viral, bacterial, and fungal infections^10^ because of their unique physicochemical characteristics, such as high surface area, nanoscale dimensions, and readily achievable surface modifications. These properties enable scientists to improve drug pharmacokinetics, control drug release, enhance drug solubility, facilitate cellular membrane passage, and enhance the bioavailability of pharmaceutics against a series of viruses such as human immunodeficiency virus, herpes simplex virus, and hepatitis B virus.^11,12^ Nanomaterials are promising tools for the diagnosis and treatment of COVID-19.

The present review systematically outlines the recent advances reported in the literature on the use of nanoparticles as effective diagnostic and antiviral treatment tools against recently mutated coronaviruses. In addition, an overview of the biological properties of all human coronaviruses is provided, with evaluation of their differences and site-specific infection of the human body. The challenges and limitations encountered by this technology are discussed. Nanotechnology offers multiple roles in combating coronavirus infections, such as nanosensors, nanobased vaccines, and smart medicine.

## 2 Human Coronaviruses: An Overview on Biological Properties

To date, seven known coronaviruses (HCoVs) have been identified that infect humans. They belong to the family Coronaviridae and include SARS-CoV-1,^13,14^ HCoV-229E,^15^ HCoV-NL63,^15^ HCoV-OC4),^16^ HCoV-HKU1,^17^ MERS-CoV,^18^ and SARS-CoV-2.^19^ The HCoV-229E and HCoV-NL63 are identified as *Alphacoronaviruses*, whereas HCoV-HKU1, HCoV-OC43, SARS-CoV1, SARS-CoV2, and MERS-CoV are classified as *Betacoronaviruses.*(20) As a coronavirus that infects humans, SARS-CoV-2 is genetically similar to SARS-CoV-1 (∼79%) and MERS-CoV (∼50%).^21^

Coronaviruses are enveloped, positively sensed, single-stranded RNA with spherical capsids (120–160 nm) that collectively resemble a crown with a solar shape.^22,23^ The CoV genome is about 26.4–31.7 kb, which is the largest among RNA viruses with guanine and cytosine contents varying from 32 to 43%.^24^ Genomic RNA acts as a mRNA (mRNA) which plays a key role in the replication of the viral genome and production of new infectious virus particles.^25^ The 5′ untranslated region (5′ UTR) and 3′ untranslated region (3′ UTR) are the regions of mRNA involved in many regulatory aspects of gene expression with a major role in RNA–RNA interactions for binding with viral and cellular proteins.^26^ A typical CoV comprises at least six-open reading frames (ORFs). Two-thirds of the genome consists of ORF1a and ORF1b, which produce two polypeptides, pp1a and pp1ab. These polypeptides are processed by viral proteases (e.g., 3-C-like protease (3CLpro), main protease (Mpro) and papain-like protease (PLpro)) for cleaving the 16 nonstructural proteins (NSPs) that are involved in genome transcription and replication.^27^ The sizes of the NSPs vary in different CoV strains.^21^

**Figure 1:**
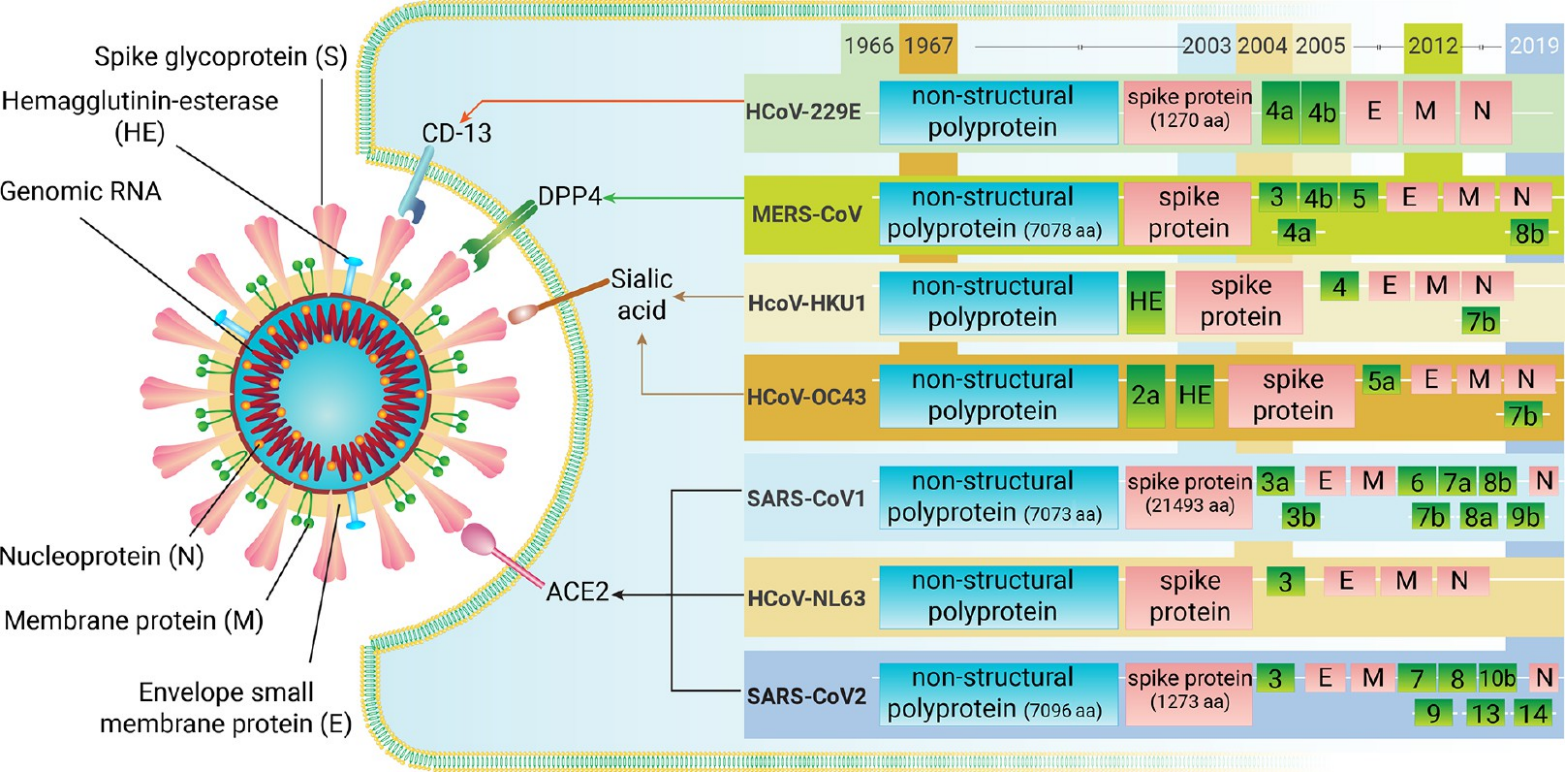
Biological properties of different types of human coronaviruses (HCoVs) that emerged over the years. In general, arrangements of the envelope (E), membrane (M), and nucleocapsid (N) proteins are different among the CoVs. The size of nonstructural proteins (NSPs) is varied in different CoVs strains. For example, 30 119 bp (7078 aa) in MERS-CoV, 29 844 bp (7096 aa) in SARS-CoV-2, and 29 751 bp (7073 aa) in SARS-CoV-1. The specific receptors used by CoVs are also different: 9-O-acetylated sialic acid is utilized by HCoV-OC43 and HCoV-HKU1, human aminopeptidase N (CD13) by HCoV-229E, dipeptidyl peptidase 4 (DPP4) by MERS-CoV, and angiotensin-converting enzyme 2 (ACE2) by HCoV-NL63, SARS-CoV1, and SARS-CoV2. Abbreviations: human coronaviruses, HCoVs; human aminopeptidase N, CD13; dipeptidyl peptidase 4, DPP4; angiotensin-converting enzyme 2, ACE2; nonstructural proteins, NSPs.

Coronaviruses have four canonical structural proteins including spike protein (S), envelope protein (E), membrane protein (M), and nucleocapsid protein (N). Besides, there are several nonstructural proteins that are encoded by ORFs 10 and 11 on one-third of the genome near the 3′ end.^28^ The S protein is a large glycosylated transmembrane protein (1160–1400 aa) that plays an essential role in the recognition of cellular receptors for infection of a susceptible cell. The size of this protein differs among the coronaviruses: 21493 aa, 1270 aa, and 1273 aa for SARS-CoV-1, MERS-CoV, and SARS-CoV-2, respectively.^21^ The E protein is a small envelope protein (74–109 aa) responsible for the assembly of virions and curving of the viral envelope.^29^ The M protein is an integral glycoprotein (250 aa), which has three transmembrane regions and interacts with other structural proteins to maintain the virion structure.^30^ The N protein is a heavily phosphorylated nucleocapsid protein (500 aa), which has a key role in encapsulating the viral genome into helical nucleocapsid within the viral particles.^31^ The arrangement of N, E, and M proteins among coronaviruses is different, as shown in Figure 1.^32,33^ The *Betacoronavirus* genus has an additional structural protein, hemagglutinin-esterase (HE, 430 aa) and a transmembrane protein that forms homodimers.^33^ The HE protein has acetyl-esterase function that is not necessary for in vitro viral replication. However, the HE protein may affect early viral infection in vivo by binding reversibly to O-acetylated sialic acids. The 3a/b and 4a/b proteins are other mature proteins responsible for various important functions in virus replication and genome maintenance.^27^

The receptors utilized by human CoVs typically include 9-O-acetylated sialic acid by HCoV-OC43 and HCoV-HKU1,^34^ human aminopeptidase N (CD13) by HCoV-229E,^35,36^ dipeptidyl peptidase 4 (DPP4) by MERS-CoV,^37^ and angiotensin-converting enzyme 2 (ACE2) by HCoV-NL63, SARS-CoV1, and SARS-CoV2.^35,38^ In addition, protease can help CoVs enter cells. For example, transmembrane protease serine 2 (TMPRSS2) and airway trypsin-like protease TMPRSS11D activate the S protein in HCoV-229E, SARS-CoV-1 and SARS-CoV-2 infections,^39^ while cathepsin L is activated in SARS-CoV and MERS-CoV.^40^ After the virus enters a susceptible cell, the genome is transcribed and translated. Replication and transcription of the coronavirus genome occur with continuation/discontinuation of RNA synthesis that is mediated by a huge replicase complex.^41^ The replicase complex is about 20 kb and contains up to 16 viral subunits along with a number of host cellular proteins.^42^ After the cellular and molecular processes, the protein is assembled on the cell membrane. Genomic RNA that buds off the internal cell membranes is converted to the mature particle forms.^43^

## 3 Mechanism of Entry of Coronaviruses into Cells

**Figure 2:**
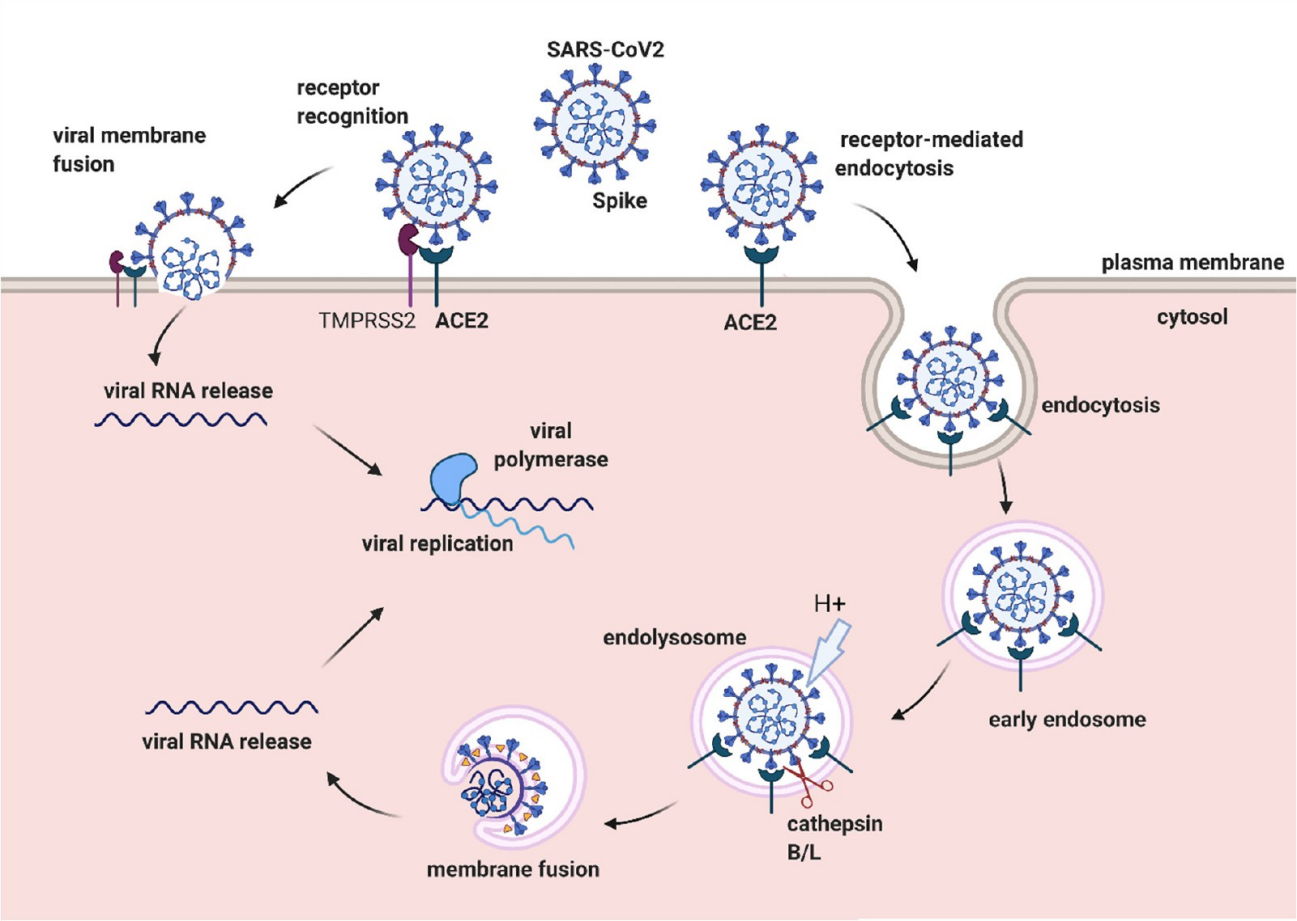
Schematic of the mechanism of entry of SARS-CoV-2 into a host cell. Binding of the SARS-CoV-2 to the cell surface is facilitated by host cellular proteins. The recognition and binding of virions occur via interaction between virion-associated spike protein and the host’s ACE2 receptor. Activation of the spike protein is mediated by the cell surface serine protease TMPRSS2, which mediates the fusion of the viral membrane with the cell plasma membrane and the release of the viral RNA into the cytoplasm of the host cells. In the absence of the cell surface proteases, after the engagement of the ACE2 receptor, entry of the SARS-CoV-2 occurs via clathrin-mediated endocytosis. During endosome maturation, the low pH activates endosomal cysteine proteases cathepsin B/L, which prime the S protein, allowing membrane fusion and release of the viral RNA from the late endosomes/lysosomes. Abbreviations: angiotensin-converting enzyme 2, ACE2.

Blocking of entry of coronaviruses into the host cell is one of the basic approaches in preventing viral infections. Because the pathogenesis of coronaviruses has not been fully understood, the precise molecular mechanism by which the virus enters a cell is unknown.^44^ Two routes are used by CoVs for entering human cells. These routes are categorized as direct delivery of the viral genome into the cytosol through fusion with the host cell membrane and through endocytosis (Figure 2).^45^

Coronaviruses enter the host cell through the interaction of their structural spike protein with cell surface receptors. The S1 subunit of the viral spike protein binds with its receptor through the receptor binding domain (RBD), after which fusion with the viral cell membrane commences through the spike S2 subunit.^46^ The ACE-2 receptor is the major receptor for entry of the SARS-CoV-1 and SARS-CoV-2 into a human host.^47^ Moreover, neuropilin-1 (NRP1) and CD147 were recently identified as host cofactors that enhancing the entry of SARS-CoV-2 via endocytosis.^48,49^ Proteolytic cleavage at the S1/S2 and S2′ sites by host cell proteases is required for the conformational changes of the S protein and for the viral fusion with the cell membranes. The cell surface serine protease transmembrane serine protease 2 (TMPRSS2) and endosomal cysteine proteases cathepsin B and L (CatB/L) are responsible for the activation of the spike proteins.^50^ After binding to the ACE2 receptor, host proteases on the cell surface mediate virus fusion at the level of the plasma membrane or with the endosomal membrane, with subsequent release of the viral genome into the host’s cytosol.^51^ Release of endocytosed virions into the cytosol is usually dependent on the pH of the endosomes, whereas direct entrance of the virions into the cytosol is pH-independent.

Recently, it has been proposed that Sars-CoV-2 employs clathrin-mediated endocytosis as the mechanism for cell entry.^52^ However, similar to the SARS-CoV and other CoVs, SARS-CoV-2 may utilize multiple pathways to gain access into the host cell cytosol.^53−57^ To date, 11 clinically approved generic drugs have been identified as potential candidates for blocking the routes of entry of SARS-CoV-2, including direct fusion with the cell membrane.

## 4 Infection of Host Organs by Coronavirus

**Figure 3:**
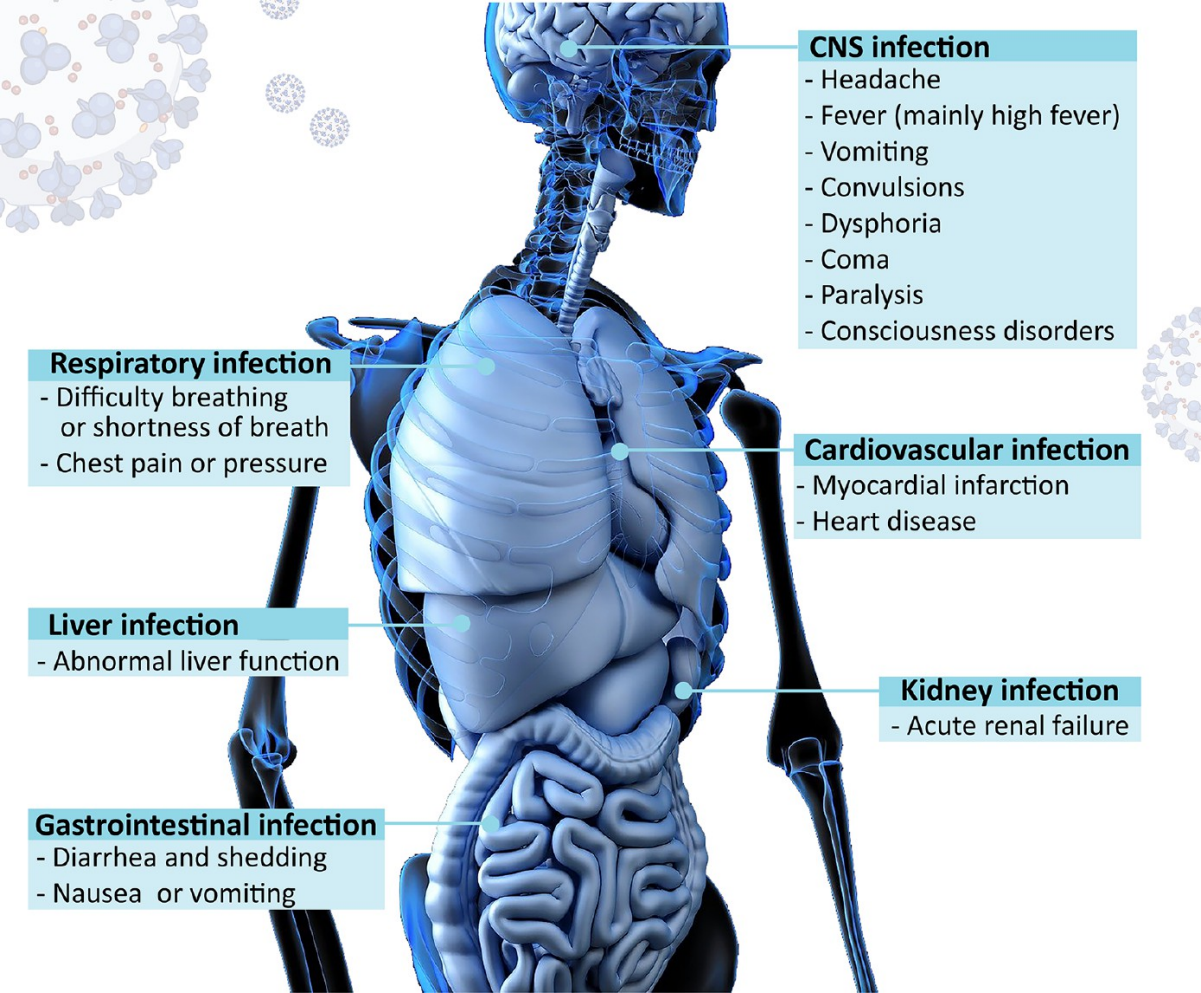
Host organs involved in coronavirus infection and the corresponding symptoms.

Although human COVs generally cause upper respiratory tract infections with relatively mild symptoms, SARS-CoV-1, MERS-CoV and the recent SARS-CoV-2 have caused severe epidemics of acute respiratory syndromes. Because viruses are cleared by the immune system, viral infections typically remain in the respiratory tract with minimal local clinical consequences.^58^ However, in some cases, viruses can evade the immune system and spread to other tissues, including the respiratory system, central nervous system, cardiovascular system, gastrointestinal system, liver, and kidney, where they induce other types of pathologies (Figure 3).^59,60^

### 4.1 Respiratory System Infection

The most common complication of coronaviruses is respiratory system infection. The clinical manifestations include fever, dry cough, dyspnea, and fatigue. Pulmonary manifestations of the recent COVID-19 pandemic have varied from asymptomatic infection to respiratory failure and death.^61^ The main receptor for the entry of SARS-CoV-1 and SARS-CoV-2, the ACE2 receptor, is heterogeneously distributed in the upper and lower respiratory tract. It is expressed at high levels in the sinonasal cavity and pulmonary alveoli, as well as on the apical side of type II alveolar epithelial cells in the lung parenchyma. This partially explains the preference of lung cells as a target for replication of these viruses.^62^ SARS-CoV-2 infection causes strong alveolar injury and acute interstitial pneumonia. The latter is characterized by macrophage infiltration, hyaline membrane formation, and alveolar wall edema and thickening.^63^ There are also pulmonary vascular abnormalities with pulmonary vessel hyaline thrombosis, hemorrhage, neutrophils, and lymphocyte infiltration. These symptoms are collectively described as diffuse pulmonary intravascular coagulopathy.^64^

Among the factors that determine a poor prognosis of the COVID-19 disease, there is the huge inflammatory over-reaction due to the excessive increase in circulating proinflammatory cytokines. The latter include interleukin (IL-1), IL-6, IL-12, interferons, and tumor necrosis factor (TNF)-α. This “cytokine storm” ultimately leads to an acute respiratory distress syndrome (ARDS), which is characterized by endothelial cell dysfunction, damage of the vascular barrier, capillary leakage, and diffuse alveolar damage.^65^

Other factors involved are enormous oxidative/nitrosative stress following the entrance of the virus, with the occurrence of apoptotic cell death and necrosis.^66^ A severe form of ARDS with low oxygen saturation levels and respiratory failure is the leading cause of mortality for SARS-CoV-2.

The molecular mechanism involved in the pathogenesis of SARS-CoV-2 is characterized by cytokine dysregulation. Accordingly, cytokine blockers such as tocilizumab, sarilumab, and siltuximab monoclonal antibodies^67^ or corticosteroids such as dexamethasone^68^ are considered promising therapeutic candidates for counteracting lung hyper-inflammation. These medications generally improve clinical outcomes.

### 4.2 Central Nervous System Infection

Detection of CoVs RNA in human brain samples indicates that these viruses are neuroinvasive and neurotropic, with the capability of causing CNS diseases.^69^ It has been demonstrated that HCoV-OC43 RNA has the potential to cause persistent infection in human CNS cells for at least one year in a murine model of acute viral encephalitis.^70^ In murine CNS, neurons were the main target of viral infection; the neurons were degenerated via programmed cell death.^71^ The S glycoprotein of the virus plays a major role in the neurodegenerative mechanism.^72^ Infections involving HCoV-229E, HCoV-OC43, SARS-COV-1, and SARS-COV-2 have been identified in various human neurological diseases, such as Parkinson’s disease, multiple sclerosis, and acute disseminated encephalomyelitis.^73−75^ To date, there have been no reports on the presence of HCoV-HKU1, HCoV-NL63, or MERS-CoV in the central nervous system of humans. However, several studies have shown that neurological symptoms are associated with HCoV-HKU1, HCoV-NL63, and MERS-CoV.^76,77^ SARS-CoV-2 was detected in capillary endothelial cells in the frontal lobe tissues obtained from the post-mortem examination.^78^ According to that report, viral infections that cause neurodegenerative diseases can impair the function of the blood brain barrier and illicit a systemic inflammatory response.^79^ The systemic inflammation triggered by coronavirus infection may cause neuroinflammatory reactions that increase susceptibility of the infected individual to neurological disorders.^58^ Infection of the central nervous system may expedite the progression of neurodegenerative diseases in at-risk individuals.

### 4.3 Gastrointestinal Infection

The relation between respiratory infection and the gastrointestinal tract has not been completely understood. Patients with respiratory infections typically have intestinal dysfunction. This is indicative of the crosstalk between the gastrointestinal tract and the lung.^59,80^ A recent case study identified SARS-CoV-2 RNA in a stool specimen, where the virus utilized ACE2 receptor for the entry into the cells.^81^ Indeed, ACE2 expression correlates with neutral amino acid transporter B0AT1 (SLC6A19) expression in the gastrointestinal tract, which increases the susceptibility of an individual to CoV infection.^82^

### 4.4 Cardiovascular Infection

Myocardial damage caused by CoV infection increases the complexity of patient treatment. Recent studies reported that MERS-CoV and SARS-CoV-2 can cause severe myocarditis and heart dysfunction.^83,84^ The mechanism of severe myocardial damage caused by CoV infection may be related to the ACE2 cell surface receptor. Indeed, ACE2 is extensively expressed both in the lung and in the cardiovascular tract. Hence, ACE2-related signaling pathways may have a key role in heart dysfunction.^85^ Other suggested mechanisms of myocardial injury include a cytokine storm that is triggered by an imbalance between type 1 and type 2 T-helper cells, and respiratory dysfunction and hypoxemia caused by SARS-CoV-2 that result in damage of the myocardial cells.^86^

### 4.5 Liver Infection

Post-mortem examination of patients infected by SARS-CoV identified the presence of a large number of virus particles in the lungs, liver vascular endothelium, and parenchymal cells.^87^ In addition, SARS-CoV-1 RNA was demonstrated in hepatocytes by reverse transcription-polymerase chain reaction (RT-PCR).^88^ Because the ACE2 receptor is abundantly expressed in the endothelial cells of liver, it has been proposed that SARS-CoV-1 utilizes this receptor for cell entry.^89^ Both liver cells and bile duct epithelial cells express ACE2 receptors.^90^ However, bile duct cells express more ACE2 receptors than liver cells. Because bile duct epithelial cells play an important role in liver regeneration and immune response,^91^ it has been suggested that liver damage that occurs in CoV patients is attributed to the damage of bile duct cells and not the virus infection.

Liver enzymes and bilirubin levels increased in patients with MERS-CoV infection, whereas albumin levels decreased.^92−96^ A more recent study reported that liver dysfunction in patients with severe SARS-CoV-2 infection was significantly more extensive than that patients with mild SARS-CoV-2 infection only.^81^ In those patients with severe SARS-CoV-2 infection, the levels of liver enzymes such as alanine aminotransferase, aspartate transaminase, and gamma-glutamyl transferase are considerably high.^97^

Patients infected with coronavirus who have other liver comorbidities such as hepatitis B virus (HBV) or hepatitis C virus (HCV) infections are more susceptible to liver damage and the manifestation of acute hepatitis. This may be attributed to the promoted replication of the hepatitis virus during CoV infection.^98^ The antibiotics, antiviral medication, and other drugs used for the treatment of CoV infection probably cause liver dysfunction.^99,100^

### 4.6 Kidney Infection

Studies have shown that CoVs (SARS and MERS-CoV) can attack the kidney and cause acute kidney injury.^49,101^ It is well shown that ACE2 receptors are not only expressed in the lung, heart, liver, and brain but are also present in the kidney.^102,103^ Thus, the virus can utilize this receptor for entry into the kidney. Patients suffering from SARS-CoV-2 infection have been reported to have a higher frequency of renal and kidney abnormalities.^104^

## 5 Diagnosis

The innate immune system provides excellent defense against viruses, otherwise primary prevention is the only alternative option. For this reason, diagnosis remains the most effective approach to control virus infection.^105^ There is growing interest in virus detection through the use of molecular-based techniques. These approaches have been classified into the amplification or nonamplification molecular-based techniques.^106^

Molecular-based techniques are more rapid and sensitive than serological techniques, either as a simple method for the manual detection of viruses or as a part of highly developed techniques.^107^ Fully automated detection systems are generally preferred in medicine. Biosafety issues and time concerns associated with the clinical usage and study of viruses are eliminated with the use of such systems. Despite all the purported benefits associated with the newly developed molecular techniques, there are still potential restrictions regarding their accuracy, sensitivity, specificity, and even reproducibility. These restrictions are mainly caused by the genetic inconsistency of viruses.^108,109^ In addition, these assays are expensive and time-consuming, requiring specific laboratory instruments as well as expert human resources.^110^ Nanomaterials with unique properties, including optical, electronic, mechanical, and magnetic characteristics, are considered attractive substrates for biomedical imaging and clinical diagnosis.^111,112^Table 1 compares the rants and raves of common virus detection methods. A wide range of nanomaterials has been proposed for virus detection. These nanomaterials include metal, silica and polymeric nanoparticles, quantum dots (QDs), and carbon nanotubes (Table 2).^113^

**Table 1:** Advantages and Cons of Common Virus Detection Methods

	technique	detection base	advantages	disadvantages	ref
basic detection	cell culture	infection test	broad spectrum; low-cost	difficulty in maintaining cell cultures; lengthy test	(114)
	electron microscopy	viral particle	broad spectrum; low-cost	require the presence of ∼106 virus particles/mL for detection; similarity of morphologies	(115)
serological detection	immunoblotting assay, neutralization assay, immunochromatographic test and complement-fixation test, enzyme-immunoassay/chemiluminescent immunoassay, radioimmunoassay, immunoprecipitation assay, hemagglutination-inhibition	viral protein	easy; low-cost	poor sensitivity; necessity for fresh reagents	(116,117)
molecular detection	polymerase chain reaction, reverse transcription polymerase chain reaction, loop-mediated isothermal amplification	viral nucleic acid	high sensitivity; easy to set up	extremely liable to contamination; not easy to quantitate results; high-skill operator required	(118)
nanobased detection	nanobiosensors	viral protein/nucleic acid	extremely high selectivity and sensitivity; high stability; fast response; portable system	pH and temperature influence the selectivity and sensitivity of biosensor	(119)

**Table 2:** Summary of Representative Engineered Nanomaterials Employed As Biosensors for Virus Detection^a^

	nanoparticles (NPs)	characteristics	target viruses	biosensor type	ref
inorganic nanoparticles	silver (AgNPs)	fluorescent properties of AgNPs introduce high sensitivity to optical-based biosensors	HBV	optical/electrochemical	(141−144)
			HIV		
			CoVs		
			KSHV		
			WNV		
			influenza		
	gold (AuNPs)	AuNPs have been used extensively for highly sensitive detection of viral diseases due to their unique optical and electrical properties	HTNV	optical/electrochemical	(145−148)
			RVFV		
			DENV		
			HEV		
			KSHV		
			IAV		
			HPV		
			HIV		
			CoVs		
	magnetic (MNPS)	controllable by an external magnet; MNPs are extensively utilized in reusable biosensor platforms	IAV	piezoelectric/electrochemical	(149,150)
			HBV		
			CoVs		
	zinc oxide (ZnO)	with piezoelectric properties, ZnO plays a main role in special sensors known as mechano-chemicals	HIV	piezoelectric/electrochemical	(151)
	copper NPs	small size and high surface-to-volume ratio of copper NPs enable them to interact closely with viruses for easy detection	HBV	electrochemical	(152,153)
			IAV		
	aluminum (AINPs)	nanoporous morphology of AINPs is the most prominent and attractive feature for designing biosensors; porous structure enhances the surface-to-volume ratio that results in an increased number of target molecules inside pores	DENV	electrochemical	(154,155)
			Ebola		
	quantum dots (QDs)	QDs are nanosize particles with unique optical and electrical properties and are powerful tools for providing rapid and sensitive virus detection to facilitate early treatment and monitoring of viral disease	HIV	optical/electrochemical	(156−158)
			HBV		
			EBV		
			CoVs		
	silica NPs	many biomolecules, such as antigen-antibodies, peptides and DNA, can be attached to the surface of silica NPs, making this platform important for bioanalytical studies	HBV	optical	(159,160)
			HPV		
organic nanoparticles	carbon nanotubes (CNTs)	CNT-based biosensors possess high selectivity and sensitivity due to their high surface area; this platform is also useful because of their ease of functionalization	HBV	electrochemical/FET	(161−163)
			HPV		
			influenza		
	graphene oxide (GO)	size controllability of GO nanosheets and changes in their oxidation level are unique features for this biosensor platform to detect specific viruses	HBV	optical/electrochemical/potentiometric	(164−168)
			HIV		
			HIV-1 rotavirus		

a Abbreviations: hepatitis B virus, HBV; human immunodeficiency virus, HIV; human papilloma virus, HPV; dengue virus, DENV; Hantaan virus, HTNV; Rift Valley fever virus, RVFV; hepatitis E virus, HEV; Kaposi’s sarcoma-associated herpesvirus, KSHV; influenza A virus, IAV; field effect transistor, FET.

According to the WHO, the current trend of CoV diagnostics is focused on the development of nucleic acid- or protein-based detection methodology for point-of-care testing (POCT).^120,121^ Nanobiohybrid platforms, containing at least one component derived from virus (e.g., nucleic acid, antibody, antigen, or structural peptide) are conjugated to various NPs.^122^ These systems rely on functioning of NPs as well as the activity of the conjugated biomolecules and/or compact multivalent probes for signal transduction.^123^ These specific NP-based probes are used in a variety of optical, electrical, and electrochemical assays for single and multiple virus detections.^124^

**Figure 4:**
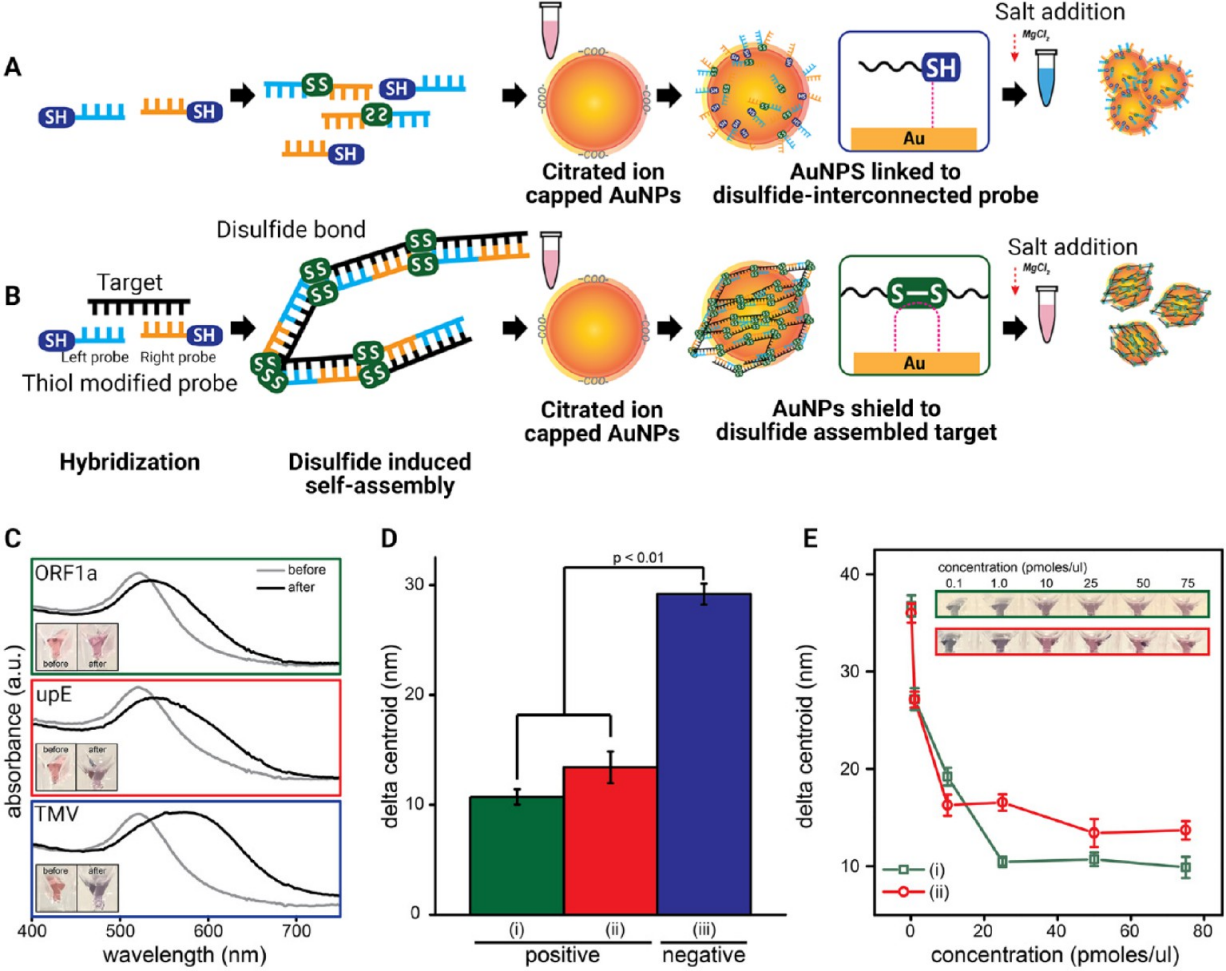
Colorimetric detection of DNA using gold nanoparticles (AuNPs): (A) Salt-induced AuNP aggregation in the absence of targets. (B) In the presence of targets, the disulfide coupling bonds induce self-assembly and prevent aggregation of the AuNPs. This results in color change that is visible to the naked eye. (C) Ultraviolet–visible light spectra of the AuNPs solution before and after adding salt in the presence or absence of disulfide-induced self-assembled targets (positive samples (open reading frames (ORF) 1a and upstream of E protein (upE)) and negative samples (tobacco mosaic virus (TMV)). (D) Average delta centroid of positive controls and the negative control at 0.1 M MgCl_2_. (E) Limit-of-detection graph of the positive control according to the target concentration. Abbreviations: gold nanoparticles, AuNPs; open reading frames, ORF; upstream of E protein, upE; tobacco mosaic virus, TMV. Reproduced with permission from ref (132). Copyright 2019 American Chemical Society.

A quantum dot-conjugated RNA oligonucleotide system has been designed for highly sensitive imaging. The system was installed on a biochip for the recognition of SARS-CoV-1 nucleocapsid (N) protein.^125^ More recently, RT-PCR was combined with lateral flow immunoassay for rapid detection of MERS-CoV.^126^ Nucleic acid testing can also be combined with the lateral flow assay. For example, a multiplex colorimetric paper-based analytical device was developed using AgNPs as a colorimetric substrate to detect the DNA associated with MERS-CoV infection.^127^ Another system was developed using self-assembled nanostructure that consisted of AuNPs and quantum dots.^128^ This platform was used as an immunosensor for the detection of Avian coronavirus (IBV) infected birds. Nanonested PCR was employed with AgNPs to distinguish between the variant and the classical strains of porcine epidemic diarrhea corona virus.^129^ In another study, a method was developed for detection of IBV using magnetoplasmonic NPs and zirconium-quantum dots conjugated with IBV antibodies.^130^ Notably, there was no reaction between the magnetoplasmonic NPs and Zr-quantum dots until the targeted virus was added.^131^ Compared with conventional analysis, this immunosensor possesses remarkable advantages, including higher sensitivity, faster analysis and accuracy comparable to enzyme-linked immunosorbent assay. In 2019, a range of signal amplifying techniques were introduced, including thermal imaging and assembly of multiple AuNPs, for improving the lateral flow readout signals for the detection of MERS-CoV (Figure 4).^132^

An immunochromatographic strip (ICS) was introduced for the detection of IBV in infected chickens based on the use of IBV-specific monoclonal antibodies against S glycoprotein and N proteins.^133^ Monoclonal antibody–colloidal gold conjugates were utilized as tracers during the preparation of ICS. The assembled ICS was identified as a specific test for IBV antigens, compared to RT-PCR.^133^ Considering that RT-PCR is an expensive technique, the AuNP-ICS method appears to have the potential for rapid detection of different IBV strains in chickens.

Lateral flow detection of SARS-CoV-2 antigen has been used to improve COVID-19 diagnosis as a point-of-care approach.^134^ In the lateral flow assays, a paper strip is coated with AuNP–antibody conjugates in the first line and with capture antibodies in the second. A urine or blood sample is placed on the strip, while the proteins of interest are placed on the membrane.^135^ The viral antigens bind to the coated AuNPs in the first line as the sample runs through the membrane by capillary action. When the antigen/AuNP–antibody complex flows through the strip, it is immobilized by the capture antibodies in the second line and a colored line appears. The color of the complex (blue) is different from the color of the NPs (red) because of plasmon effect. Although this kind of assay shows 100% specificity for IgM and IgG, the clinical sensitivity and accuracy are different (57 and 69% for IgM, and 81 and 86% for IgG, respectively). Detecting both IgM and IgG yields a clinical sensitivity of 82%.^135^

**Figure 5:**
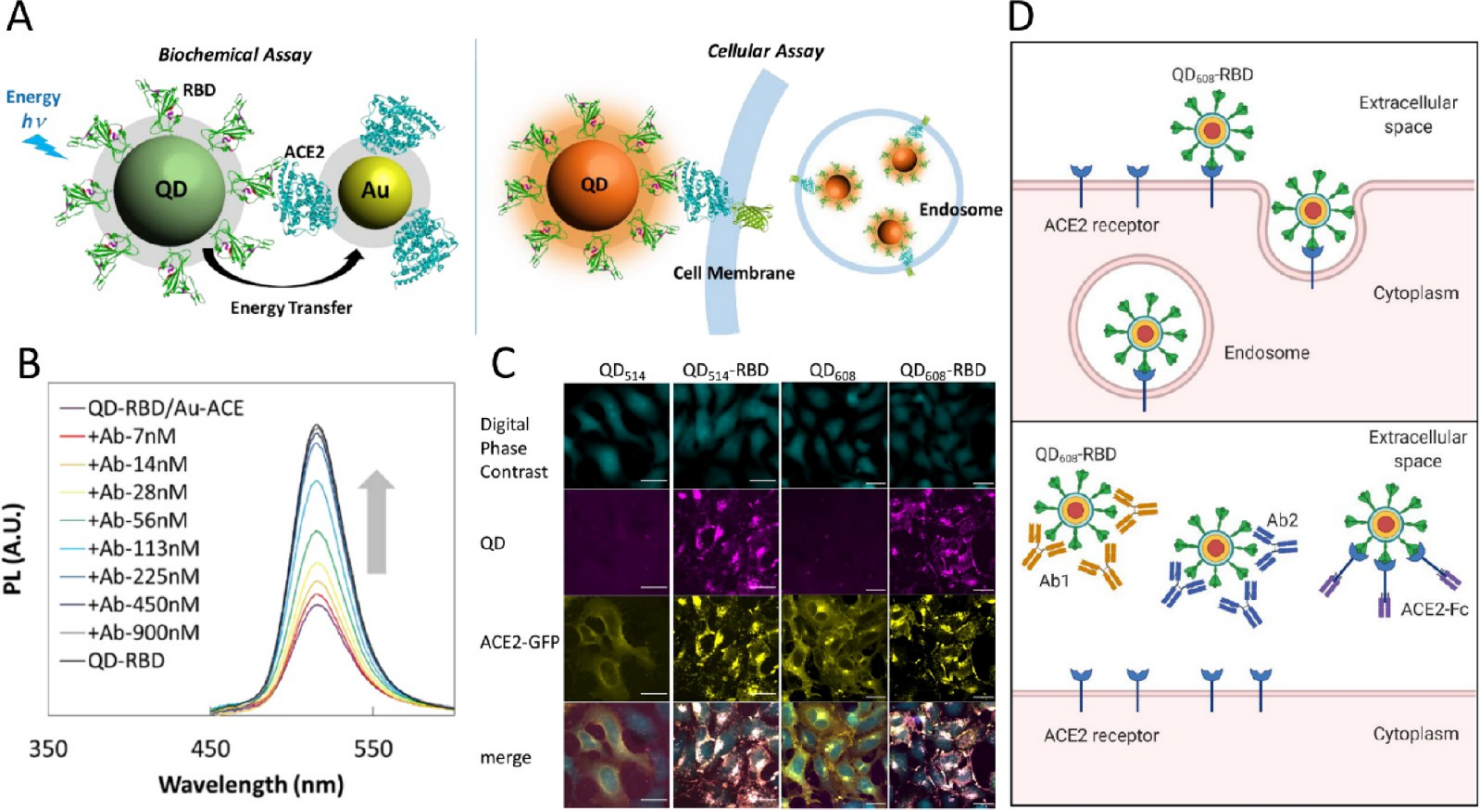
(A) Schematic of the biochemical assay using energy transfer from quantum dot-conjugated Spike Protein-RBD domain (QD-RBD) to AuNP-ACE2 (top left) and the cellular assay using QD-RBD interaction with ACE2 (with or without green fluorescent protein (GFP) modification at the end of the C-terminal) on the cell membrane (top right). (B) Evaluation of the efficacy of neutralizing antibody Ab1 that is specific for SARS-CoV-2, showing the fluorescence recovery of QD_514_-RBD in the presence of neutralizing antibody Ab1. (C) In vitro live imaging shows that QD-RBD domain induces the translocation of ACE2 and is internalized into cells, (D) Schematic of the QD-RBD internalization via receptor-mediated endocytosis and inhibition using antibodies Ab1, Ab2, and ACE2-Fc. Abbreviations: angiotensin-converting enzyme 2, ACE2; gold nanoparticles, AuNPs; quantum dot, QD; green fluorescent protein, GFP. Reproduced with permission from ref (136). Copyright 2020 American Chemical Society.

An energy transfer system has recently been developed using recombinant spike protein receptor binding domain (RBD) conjugated to fluorescent quantum dots, AuNPs, and cells with green fluorescent protein tagged ACE2 receptors (ACE2-GFP) for facile monitoring of viral spike protein–ACE2 interaction (Figure 5).^136^ In that study, fluorescence of the quantum dots was quenched upon their binding with AuNPs in the vicinity. Fluorescence was recovered by neutralizing SARS-CoV2 antibodies that compete with ACE2–AuNPs or blocking the binding of quantum dot–spike protein RBD to the ACE2–AuNPs. The in vitro bioimaging results demonstrated the potential ability of quantum dot–RBD internalization via dyamin/clathrin-dependent receptor-mediated endocytosis, with high affinity to the ACE2 extracellular domain. This platform is a promising biosensor for facile, rapid, and high-throughput cell-based screening of SARS-CoV-2 infection.

**Figure 6:**
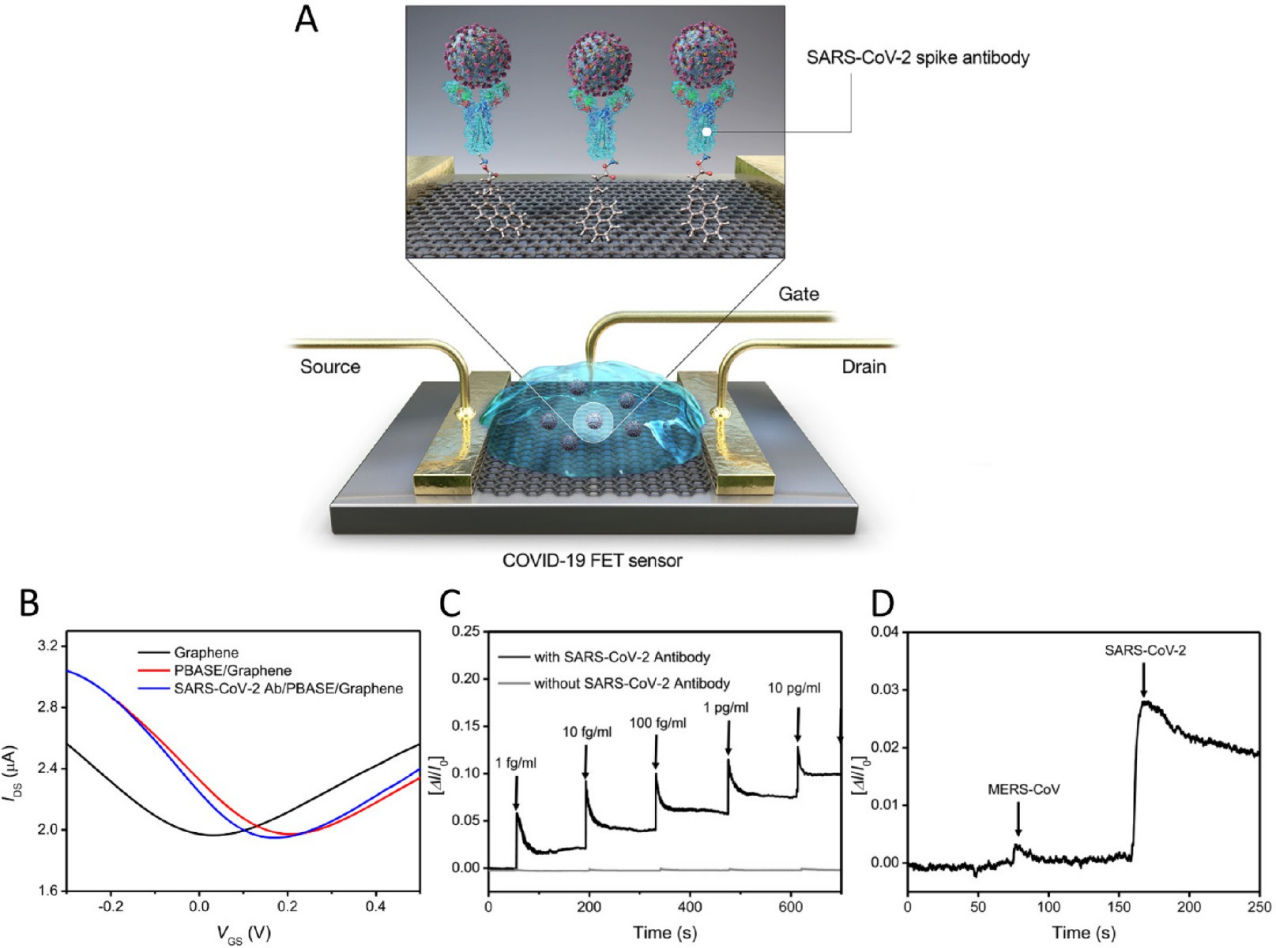
Operation procedure of the SARS-CoV-2 field effect transistor (FET) sensor. (A) Graphene is used as the sensing material. The SARS-CoV-2 spike antibody is conjugated onto the graphene sheet via 1-pyrenebutyric acid *N*-hydroxysuccinimide ester, which is an interfacing molecule and probe linker. (B) Transfer curves of the SARS-CoV-2 FET sensor in steps of the antibody conjugation (VDS = 0.01 V). (C) Real-time response of the FET-biosensor toward SARS-CoV-2 antigen protein in phosphate-buffered saline. (D) Elective response of the COVID-19 FET sensor toward target SARS-CoV-2 antigen protein and MERS-CoV protein. Abbreviations: field effect transistor, FET. Reproduced with permission from ref (137). Copyright 2020 American Chemical Society.

Another research group created an advanced field-effect transistor (FET) biosensor platform based on graphene sheets with a specific antibody against SARS-CoV-2 spike protein (Figure 6).^137^ This biosensing platform could recognize surrounding alteration on their surface and provide ultrasensitive sensing and low-noise detection. In addition, it could distinguish the SARS-CoV-2 antigen from the MERS-CoV antigen. It is a potential device for rapid and highly sensitive detection of CoVs from clinical samples.

Metal NPs (e.g., Au, Zr and Ag NPs, as well as MoS_2_ nanosheets) and quantum dots were employed for the detection of a range of coronaviruses.^138^ Conjugating nanomaterials with colorimetric, electrochemiluminescence, immunosensing, photoluminescence, and chiroimmunosensing have also been considered as potential substrates for the detection of coronaviruses. Electrochemical devices appear to be a good alternative for the detection of new strains of coronaviruses because of their superior ability to combine with nanomaterials.^138^ Using nanomaterials in this aspect decreases the time of analysis and increases sensitivity. This strategy opens new vistas in designing better systems with higher performance in the future.

Microfluidic devices incorporated as organ-on-a-chip are considered another point-of-care system.^139^ These devices consist of a palm-sized chip fixed with the reaction chambers and micrometer-sized channels. The chip is made of various materials, including polymers, glass, or papers. The device mixes and separates liquid samples by capillary, vacuum, or electrokinetic forces.^139^ Microfluidic devices have benefits such as portability, miniaturization and the use of small sample volume for rapid detection. For example, a smartphone-based point-of-care microfluidic platform has been developed. The system was fabricated with ZnO nanorods and polydimethylsiloxane (PDMS) to detect antibodies against specific infections such as human immunodeficiency virus (HIV) infection through colorimetric detection.^140^ This platform showed 100% clinical sensitivity and 87% specificity for HIV detection in 96 patients in Rwanda. Microfluidics may be modified further for the detection of coronavirus RNA or protein.

## 6 Therapy

### 6.1 Nanomaterials to Combat Coronaviruses

Nanomaterials have been introduced as antiviral agents or drug delivery platforms for combating CoV infections.^169^ In 2014, a research group patented a mixture of silver colloid, titanium dioxide (TiO_2_) NPs and a dispersion stabilizer with antibacterial, antifungal, and antiviral behavior.^170^ The platform offers antiviral activity against CoVs such as porcine epidemic diarrhea virus (PEDV) and swine transmissible gastroenteritis virus (TGEV). When the platform concentration is diluted by 1000-fold, virus growth is inhibited at a rate of 99.9 and 93.0% for PEDV and TGEV, respectively. This activity was reliant on the platform concentration, which means that the usage dose has to be in tune with the virus in which the platform is designed to inhibit.^170^

In the same year, the induced immune responses of four silver nanoconjugates on TGEV-infected swine testicle cells were investigated.^171^ These nanomaterials included AgNPs, two Ag nanowires with mean lengths of 60 and 400 nm, and silver colloids. Silver NPs and the two types of Ag nanowires protected the testicle cells against TGEV infection and reduced the number of apoptotic cells. In contrast, the silver colloids were not capable of inhibiting cellular entry by TGEV.^171^ Graphene oxide–silver (GO–Ag) nanoconjugates that possess antiviral activities against nonenveloped and enveloped viruses were developed by other researchers.^172^ Different dilutions of GO–Ag solution were incubated with diluted solutions of feline coronavirus. The supernatant was analyzed using a virus inhibition assay after removing the GO-Ag pellets. The GO-AgNPs were able to detect nonenveloped and enveloped viruses by binding of the AgNPs to the negatively charged sulfur groups of the viral proteins, whereas pristine GO inhibited only enveloped viruses at noncytotoxic concentrations.^172^

A diphyllin-based therapeutic device was developed for the treatment of feline infectious peritonitis (FIP) caused by feline coronavirus.^173^ Diphyllin is a vacuolar ATPase required for endosomal acidification inhibition in Felis catus whole fetus-4 cells. The inhibitory behavior of diphyllin against FIP was enhanced by generating a diphyllin nanocarrier with poly(ethylene glycol)-*block*-poly(lactide-coglycolide). Diphyllin NPs demonstrated antiviral activity; even a high dosage of the NPs was tolerated by mice.^173^ Although this system was not a candidate for preparing vaccines, the study verified the efficacy of nanoformulations against coronaviruses.

**Figure 7:**
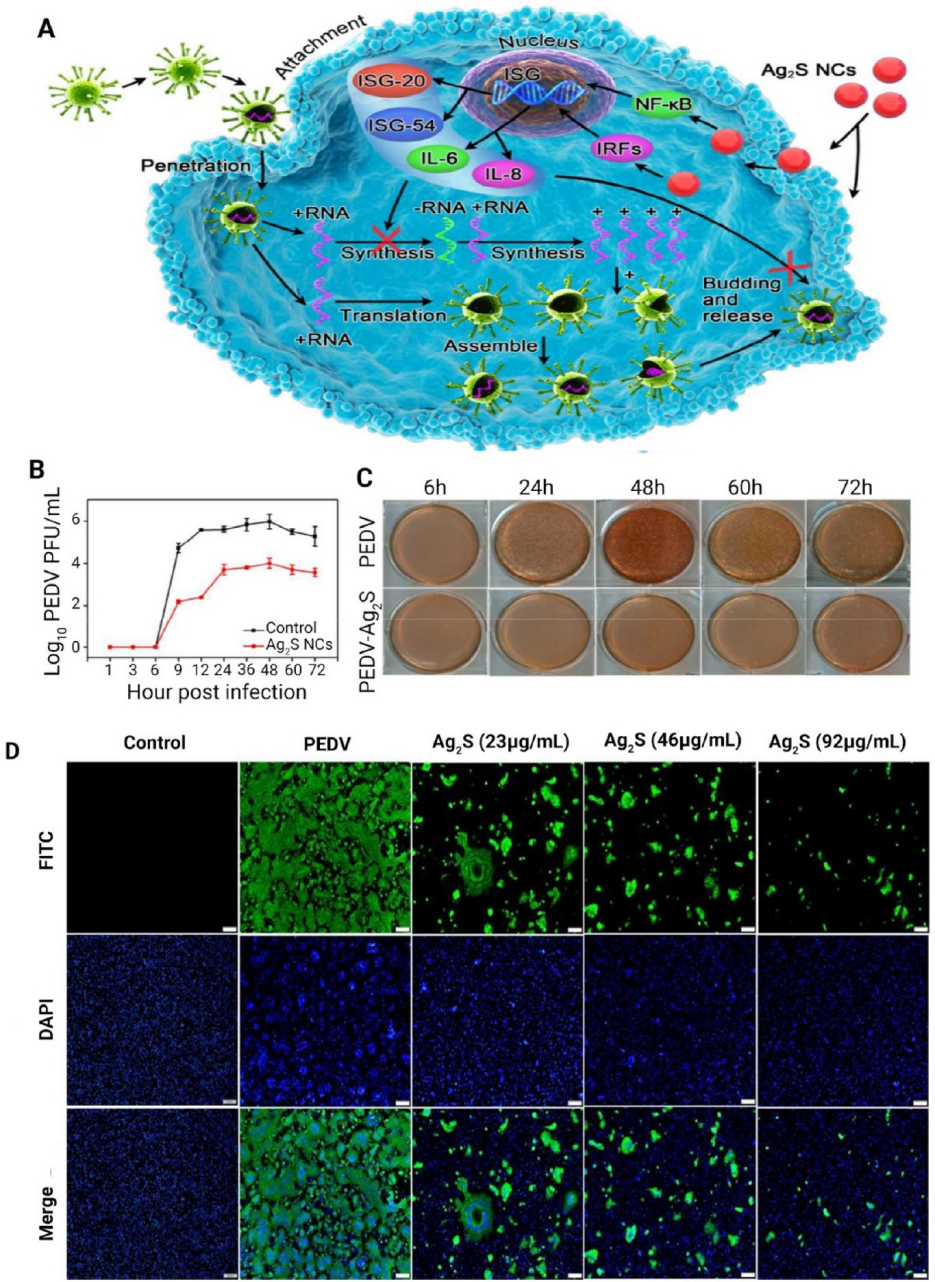
(A) Schematic of the antiviral mechanism of Ag_2_S nanoclusters against viruses, including four consecutive steps of attachment, penetration, replication and budding. Treatment with Ag_2_S nanoclusters inhibits the synthesis of viral negative-strand RNA and prevents viral budding. The activation of interferon-stimulated genes and the up-regulation of pro-inflammatory cytokines play a key role in the inhibitory effect of Ag_2_S nanoclusters. (B) Growth curves of porcine epidemic diarrhea virus (PEDV) with/without treatment with Ag_2_S nanoclusters. (C) Plaque reduction assay after neutral red staining. Pictures were taken 2–3 days after infection. (D) Immunofluorescence assay of PEDV-infected cells with/without treatment with different concentrations of Ag_2_S nanoclusters (bar: 100 μm). Abbreviations: porcine epidemic diarrhea virus, PEDV. Reproduced with permission from ref (175). Copyright 2018 American Chemical Society.

Another nanoplatform was developed using *N*-(2-hydroxypropyl)-3-trimethyl chitosan (H-HTCC) to produce nano/microspheres (NS/MS) for adsorbing coronaviruses.^174^ The copy number of viral RNA decreased when H-HTCC-NS/MS was added to the viral suspensions. The result is indicative of a good correlation between virus concentration and the amount of added biomaterial.^174^ In another novel therapeutic approach, Ag_2_S nanoclusters were fabricated for restraining the proliferation of PEDV in treated Vero cells (Figure 7).^175^ The Ag_2_S nanoclusters were capable of inhibiting the synthesis of negative-strand RNA and preventing viral budding. The Ag_2_S nanoclusters regulated the expression of interferon-stimulating genes as well as the production of pro-inflammation cytokines. This resulted in the protection against PEDV infection.^175^

Although a lot of studies illustrated the antiviral activities of nanomaterials against coronaviruses, further investigation is needed to develop antiviral nanomedications against SARS-CoV, MERS-CoV, and SARS-CoV-2.

### 6.2 Nanobased Gene Therapy of Coronaviruses

**Figure 8:**
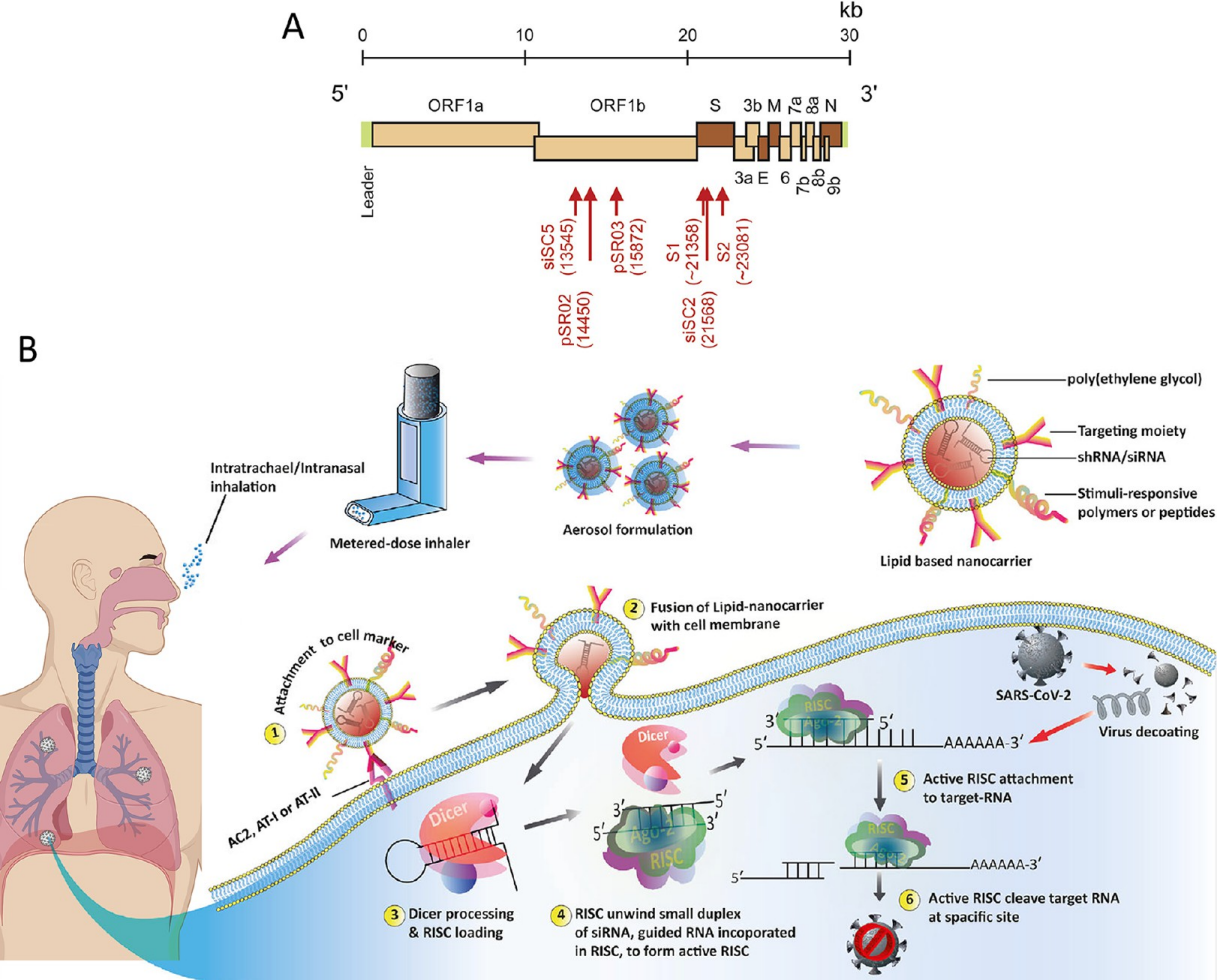
(A) Genome of SARS-CoV with targeted sites by RNAi activators. Reproduced with permission from ref (177). Copyright 2015 Elsevier, (B) Schematic of the proposed SARS-CoV-2 treatment through the use of multifunctional nanocarriers that deliver antiviral siRNA into the respiratory system to combat viral infection. Reproduced with permission from ref (200). Copyright 2020 Wiley.

Ribonucleic acid interference (RNAi) mediated by small interfering RNA (siRNA) is an effective strategy to inhibit the replication of RNA viruses. Antiviral siRNA therapy offers several advantages compared to conventional antiviral drugs and vaccines. These advantages include rapid action with high specificity and efficacy at different viral stages, the use of a less amount of siRNA to reduce viral RNA, and high homology of siRNA with cognate viral RNA.^176^ Therapy based on RNAi is a potentially promising approach to overcome SARS-CoV-2 infection. In this regard, accurate characterization of the coronavirus genome enables rapid development of effective therapeutic anti SARS-CoV-2 RNAi activators.^177^ Because the genomic sequences of SARS-CoV and SARS-CoV-2 have high homology (∼79% at the nucleic acid level), the results derived from SARS-CoV may be extrapolated to SARS-CoV-2 (Figure 8A).^178^

Several recent studies have found that RNAi is effective against SARS-CoV.^179^ A research group reported that the use of expression cassettes (plasmid-mediated siRNAs) that produced six antiviral RNAi activators could target specific sites of the viral genome. Pretransfection of Vero cells with the siRNA-expressing plasmids pSR02 and pSR03 prior to the infection of those cells with SARS-CoV resulted in blocking the replication of the ORF1b sequence of the virus genome.^180^ Targeting the S sequence effectively inhibit viral infection and replication because the S gene is a good target in SARS-CoV.^181^ RNAi activators that target both S and ORF1b regions of the viral genome have been investigated as the potential drug candidates.^182,183^ Based on these valuable results derived from the use of RNAi against SARS-CoV, gene therapy via RNAi may revolutionize the treatment of COVID-19.^184^ The therapeutic potential of RNAi in combating MERS-CoV has been investigated by using two siRNAs, Smad7-1 and Smad7-2, to knockdown MERS-CoV in both human lung and kidney cell lines. It was found that Smad7 effectively inhibited viral replication and infection in host cells.^101^

Although specific targeting of the viral genome sequence is the strength of antiviral siRNA therapy, targeted delivery of siRNA into a cell with inadequate endosomal escape is another potential approach.^185^ Application of siRNA is typically hampered by rapid enzymatic degradation of the siRNA, fast clearance and inability of SiRNA in entering cells.^186^ These challenges are mostly due to unstable negatively charged siRNA bases that stimulate unwarranted immune response, and random insertion of the siRNA into chromosomes that results in gene dysfunctions.^187^ These restrictions may be overcome by using nontoxic, biocompatible nanocarriers prepared from polymers, lipids, hybrid (polymer/lipid) NPs, nanohydrogels, silica, dendrimers, iron oxide NPs and AuNPs.^188−190^ Among these, lipids and polymers are considered promising platforms for siRNA delivery because of their highly biocompatible and biodegradable nature. For example, poly(lactic acid), polycaprolactone, poly(glycolic acid) and their copolymers have been approved by the United States Food and Drug Administration for targeted siRNA delivery in vivo.^187,191,192^

Lipid-based NPs, including solid-lipid NPs, nanostructured lipids, and liposomes, are also suitable for the preparation of siRNA delivery systems.^193^ Nanocarriers preserve the encapsulated siRNA from degradation by serum nucleases, prolong their circulation and promote their access to destined sites.^194^ Polycationic lipids or polymers maintain their low endosomal pH by increasing influx of protons and water. This causes the endosomes to rupture and release the loaded therapeutics into the cytosol.^195^ Delivery of antiviral siRNA through commercially available cationic lipid structures such as oligofectamine, lipofectamine (Invitrogen), lipofectin, TransIT TKO (Mirus), and RNAifect (Qiagen) have demonstrated promising results.^195^ Poly(lactic-*co*-glycolic acid) (PLGA), lipid, and polymer–lipid nanocarriers are suitable for loading of inhalable antiviral siRNA as well as for aerosol-based pulmonary delivery of antiviral siRNA.^196^ Cholesterol-conjugated lipid nanoparticles (LNPs) have also been developed for the delivery of an mRNA vaccine against SARS-CoV-2.^197^ Histidine-lysine copolymer and spermine-liposome conjugate-based nanocarriers have also been approved for siRNA delivery to target specific sequences in the SARS-CoV genome.^198^ Coronavirus-infected mice that were treated with intranasally delivered nanoformulated antiviral siRNA showed very positive effects. Considering these successful achievements, the use of cationic-liposomal encapsulated antiviral-siRNA and their aerosol formulation appears to be a reasonable treatment for SARS-CoV-2 infection.^199^ A lipid/polymer-based nanocarrier modified with functional molecules (i.e., antibodies or aptamers) was effective in delivering siRNA to target sites through intranasal or intratracheal administration via an inhaler (Figure 8B).^200^ The use of antibodies against alveoli-specific surface markers type-I and II (AT-I and AT-II) is a good alternative for functionalization of nanocarriers and the subsequent delivery of therapeutic siRNA to lung cells and other organs that express these markers. The surface of nanocarriers may also be functionalized with polyethylene glycol and pH-sensitive histidine-lysine peptide for prolonged circulation and endosomal release of siRNA to the cytosol for inducing the RNA interference pathway.^200^ Activation of the RNA interference pathway results in cleavage of the viral RNA at the targeted site, which is critical for combating viral infection.

### 6.3 Nanobased Immunotherapy against Coronaviruses

Immunotherapy-based NPs have gained attention as a highly effective treatment modality for combating infectious diseases. However, there are still challenges associated with increasing therapeutic efficiency and reducing side effects. Understanding the function of the immune system against infection and the possible approaches to modulate immunity are essential steps toward the design of effective immunotherapy.

#### 6.3.1 Immune Responses against Coronaviruses

The immune responses to CoVs include innate and adaptive immunity. When CoVs encounter the first line of immune defense (i.e., mucus and ciliated cells), the pathogen-associated molecular patterns (PAMPs) on the virus surface alert the innate immune cells to the presence of the invading molecule. This results in the release of type I interferons (IFN-α/β).^201^ In the event of an acute infection, other immune cells, including natural killer (NK) cells, alveolar macrophages, monocytes, and neutrophils, are activated. This produces a large amount of pro-inflammatory cytokines (IFN, tumor necrosis factor (TNF)-α, interleukin (IL)-1β, and IL-6), resulting in a condition known as the cytokine storm that severely impairs the respiratory epithelial cells.^5^ The innate immune cells use pattern recognition receptors (PRRs) such as retinoic acid-inducible gene I-like receptors, Toll-like receptors, and nucleotide-binding and oligomerization domain-like receptors to detect PAMPs and generate an appropriate immune response.^202^ Subsequent interactions between PAMPs and PRRs stimulate phagocytosis by macrophages and dendritic cells and induce intracellular molecular pathways to express pro-inflammatory cytokines (i.e., type I interferons (IFN-I), IFNα/β; and type II interferon (IFN-II), IFN-γ) and chemokines (i.e., CCL-2 and CXCL-10). The IFN-I blocks the replication of viruses through multiple pathways.^203^ The infected cells that express major histocompatibility complex I induce NK cells to produce IFN-γ and stimulate apoptosis via antibody-dependent cellular cytotoxicity.^204^

**Figure 9:**
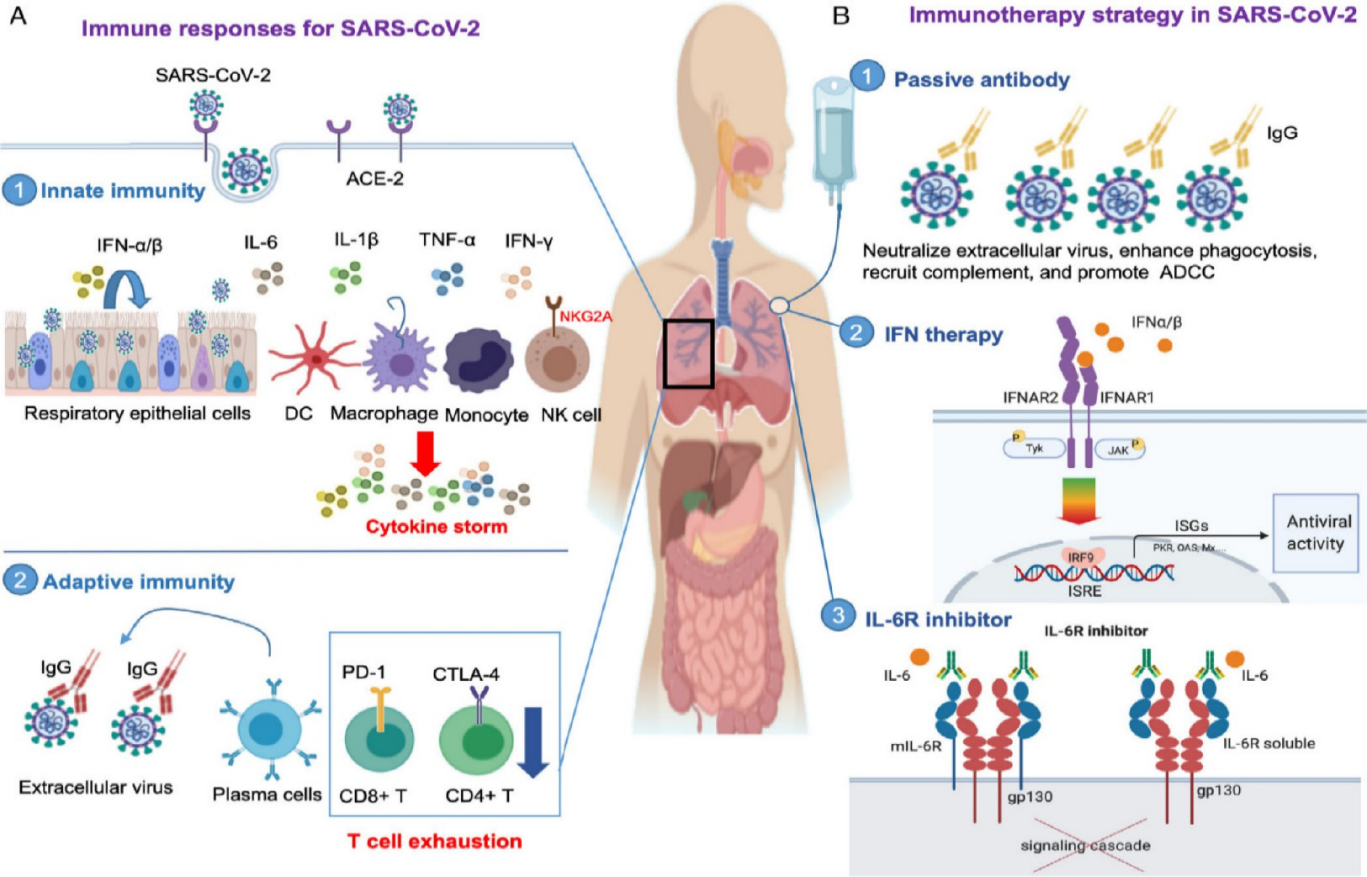
Immune responses and immunotherapy strategy in SARS-CoV-2 infection. (A) Immune response to SARS-CoV-2 involving innate and adaptive immunity. (B) Immunotherapy strategy including passive antibody therapy, interferon α/β, and IL-6 receptor (IL-6R) inhibitor. Abbreviations: IL-6 receptor, IL-6R. Reproduced with permission from ref (215). Copyright 2020 Wiley.

In SARS-CoV-2 infection, extensive production of antibodies was observed along with reduction in CD4^+^/CD8^+^ T cells.^205,206^ In this infection, macrophages and dendritic cells have an essential role in mounting specific immune responses. These cells remove virus particles through phagocytosis and IFN-I secretion, with subsequent priming of the adaptive immune responses.^207^ In additions, IFN-I inhibits the replication of viruses through upregulation of interferon stimulated genes, including protein kinase R (PKR) and 2′-5′-oligoadenylate synthase (OAS)/RNase L.^203^ These important components of the protein synthesis machinery block the synthesis of proteins via phosphorylation of OAS/RNase L and eukaryotic initiation factor 2 subunit-α (eIF2α), resulting in degradation of the viral ssRNA and impairment of viral replication.^208^ The IFN-I promotes CD8^+^ T cell priming, induces B cell activation and antibody production, and eventually stimulates NK cells and macrophages to halt viruses. Several studies have reported that MERS-CoV expresses NS4a protein to block the activation of PKR and OAS/RNase L in the innate immune responses.^209−211^ The response of the immune system to SARS-CoV-2 infection is shown in Figure 9A. Researchers suggested that using IFN-α as a pretreatment approach prior to infection with SARS-CoV could induce the expression of IFN-related genes and signaling pathways.^212^ An in vitro study reported that IFN-α could restrain SARS-CoV infection.^213^ A more recent study also indicated that pretreatment of cells with IFN-I resulted in a significant decrease in SARS-CoV-2 replication. These initial findings suggest that IFN-1 possesses antiviral activity against SARS-CoV-2.^214^ However, more clinical trials are required to validate these findings.

#### 6.3.2 Immunotherapy Strategies against Coronaviruses

Humoral immunity is crucial for inhibiting viral infection through the activation of B cells for antibody generation.^216^ Antibodies recognize and mediate the killing of the virus-infected cells via several pathways, including phagocytosis, opsonization, neutralization, and activation of the classical complement pathway, as well as mediating antibody-dependent cellular cytotoxicity.^217^ As such, the virulence of virus and the host immune response should be balanced to successfully overcome the viral infection. Although the host’s inflammatory responses in the early stages of infection is essential, the severe inflammatory responses at the late stages of the viral infection aggravate the clinical manifestations.^218^ For this reason, immunotherapy strategies that enhance viral clearance and minimize the hyper-inflammatory responses should be used to overcome coronaviruses infection.^219^ Immunotherapy against SARS-CoV and MERS-CoV infections is classified into three approaches: passive antibody therapy, interferon α/β and IL-6 receptor inhibition (Figure 9B).^215^

Passive antibody therapy includes the administration of antibodies from recovered patients to new patients involved with the same infection.^220^ Neutralizing antibodies may be isolated from individual convalescent plasma or developed as monoclonal antibodies through immortalizing B-cell repertoires of the convalescent plasma.^221^ Several issues should be regulated to improve the efficacy of passive antibody therapy. These issues include administered antibody titer, plasma administration time, and accurate convalescent plasma screening for blood-borne pathogens.^220^ The use of monoclonal antibodies is preferred in comparison with the other approaches in blocking the attachment of viruses. This because of the unique properties of monoclonal antibodies, including purity, specificity, low risk of blood-borne pathogen contamination, and safety.^222^ Monoclonal antibodies comprising different polyclonal antibodies are capable of recognizing different epitopes on the viral surface and holds promise in overcoming virus infection. Targeting the S protein as the key neutralizing antibodies inducer has also been considered for the treatment of SARS-CoV-2.^223^

The use of IFNs may overcome viral infection by promoting the expression of interferon stimulated genes that encode antiviral proteins and cytokines.^224^ Such antiviral proteins exert antiviral effects by either the hindering viral replication or inducing the adaptive immune system. According to reported experimental investigations, IFNα and IFNβ possess potent antiviral activities that restrict SARS-CoV and MERS-CoV replication.^225,226^

Cytokines are other potential targets for efficient immunotherapy of coronaviruses. Among the cytokines, IL-6 is considered more important in the treatment of SARS-CoV-2. This is because overexpression of IL-6 is associated with the severity of inflammatory cytokine storm.^227^ It has been proposed that targeting of IL-6 and its receptor (IL6R) through the use of immunosuppressive drugs such as tocilizumab and chimeric monoclonal antibody such as siltuximab can overcome cytokine storms and reduce the clinical manifestations in SARS-CoV-2 patients.^228^

The use of the described immunotherapy approaches, alone or in combination with other drugs, has been proposed for treating patients with SARS-CoV-2 infection.^229^ Notably, all immunotherapy efforts against SARS-CoV-2 mostly involve the use of polyclonal antibody via plasma therapy, polypeptide hormone for T cell maturation, neutralizing antibodies, ACE2 immunoadhesin, immunoglobulins, and monoclonal antibody against IL-6.^230^ In spite of the extensive attempts in the development of monoclonal antibody-based passive immunotherapy for combating CoV infections, no monoclonal antibody is available to date. The major limitation is that large-scale production of monoclonal antibodies is difficult, expensive, and time-consuming.^231^ Designing and developing advanced platforms and materials are essential in providing immunotherapy at a reasonable cost in a short time period.

Nanoparticle formulations are promising platforms for overcoming the hurdles associated with immunotherapy.^232^ For example, nanoparticulate forms of antigens and other immunomodulatory agents can modulate the function of immune components through enhancing multivalent receptor cross-linking, regulating intracellular processing, inducing cytosolic delivery, targeting the innate immune system, and reducing the toxicity associated with immunomodulators.^233^ In additions, nanomaterials have the potential to incorporate several antigens on their surface for more effective activation of the immune system. Thus, nanomaterials are not only therapeutic carriers but may possess immunomodulatory properties themselves, acting as potential immune adjuvants. To date, an extensive range of nanomaterials such as dendrimers, liposomes, carbon nanotubes, polymer-based materials, and inorganic NPs have been investigated as potential platforms for immunological applications.^234^ Nanomaterials such as PLGA and liposomes can activate CD8^+^/CD4^+^ T cells and promote antigen cross-presentation for effective antigen delivery.^235,236^ Moreover, inorganic NPs such as AuNPs can interact with dendritic cells, promoting the expression of pro-inflammatory cytokines (i.e., IL-1, IL-6, IL-12, IFN-α, and TNF-α), and the down-regulation of anti-inflammatory factors (i.e., transforming growth factor (TGF)-β1 and IL-10).^237,238^ Gold nanoparticles also activate T cells-related immune responses and increased the phagocytic activity of dendritic cells. Despite the progress of experimental application of nanomaterials in immunotherapy, there are relatively few fundamental investigations on the use of NP-based immunotherapy against CoVs.

### 6.4 Nanobased Vaccines against Coronaviruses

Because of their specificity and capacity to induce immune memory, vaccines are the preferred defense tools against infectious diseases, compared to chemotherapeutical drugs.^239^ Some of the current vaccines utilize either delivered or expressed viral proteins to induce neutralizing antibodies against CoVs. These antibodies inhibit viral entry by binding to the M, E or S proteins of CoVs.^240^ The use of nanobased therapeutic agents against different types of CoVs has been perceived as a potential solution based on the immunostimulatory effects of NPs.^241^

Gold nanoparticles conjugated with TGEV were used for stimulating the protective immune response against CoV in immunized mice and rabbits.^242^ The use of this antigen-colloidal gold complex resulted in the activation of macrophages and immunity against TGEV, with induction of IFN-γ production and higher titers of neutralizing antibody in vaccinated animals. Proliferation of T cells was amplified ten-fold following immunization with the antigen-colloidal gold complex, compared with the free antigen response. On the basis of this result, virus-conjugated AuNPs were suggested as a potential antiviral vaccine.^242^

**Figure 10:**
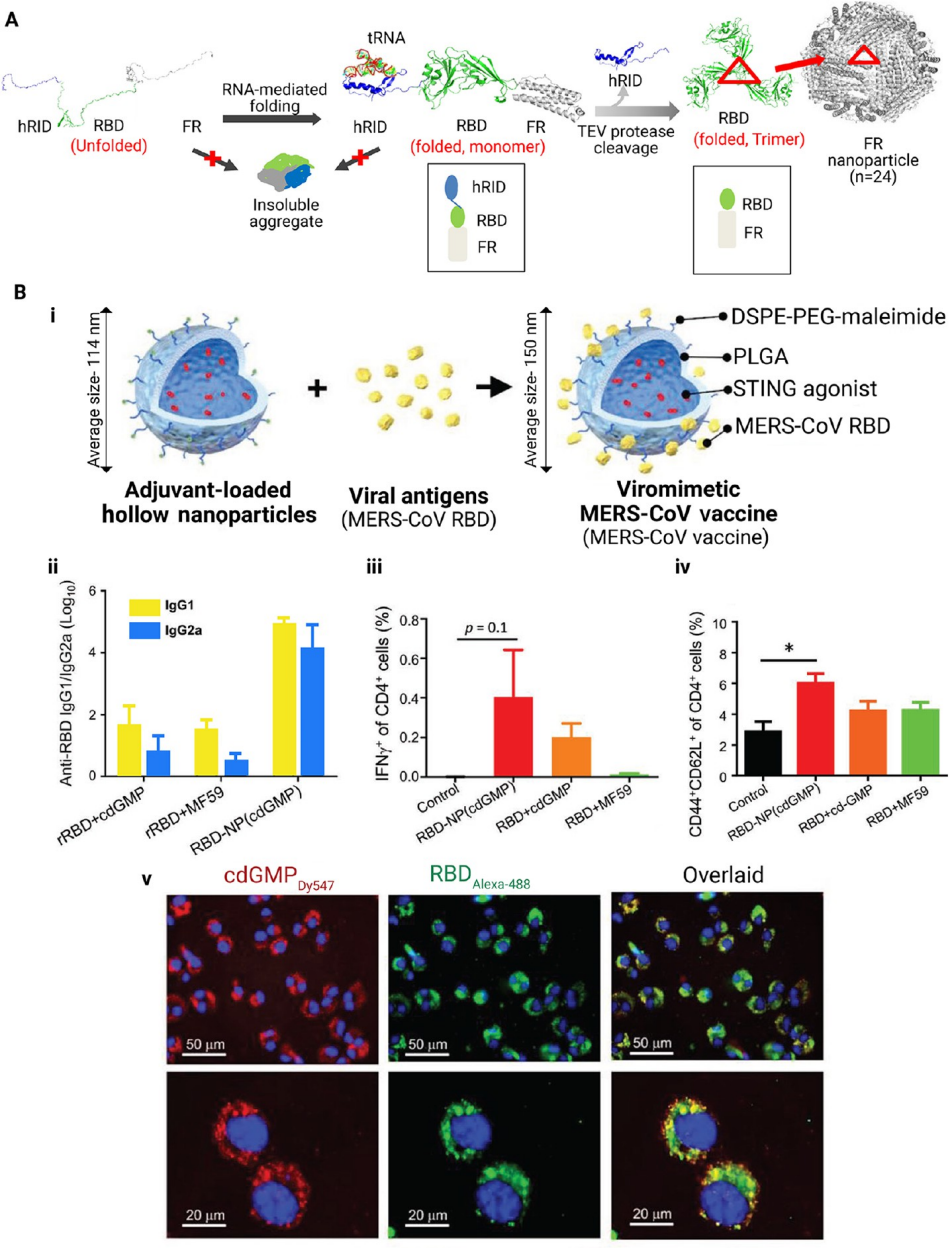
(A) Schematic of Middle East respiratory syndrome-coronavirus receptor-binding domain (RBD) nanoparticles (MERS-CoV RBD-FR NPs) using the chaperna-mediated hRID fusion partner. The hRID facilitated folding of the aggregation-prone RBD-FR through interaction with RNA. The monomer of RBD-FR forms a properly folded trimeric structure by cleaving hRID with tobacco etch virus (TEV) protease. Eight trimers assembled into MERS-CoV-like NPs. Red triangles indicated the RBD trimer on the FR NPs. Reproduced with permission from ref (243). Copyright 2018 Frontiers. (B) Schematic of the preparation of viromimetic NP vaccine: (i) Hollow poly(lactic-*co*-glycolic acid) (PLGA) NPs with encapsulated adjuvant and surface maleimide linkers were prepared. Recombinant viral antigens were conjugated to the surface of NPs via thiol-maleimide linkage. (ii) MERS-CoV RBD-specific IgG1 and IgG2a titers in immunized mice on day 35 postvaccination (*n* = 6). (iii) CD4^+^ T-cell responses against MERS-CoV RBD in immunized mice were determined by intracellular cytokine staining on day 7 after boosting (*n* = 3). (iv) Frequency of central memory (CD44^+^CD62L^+^) CD4^+^ T cell in the draining lymph nodes of immunized mice, 28 days after boosting (*n* = 3). (v) Cellular distribution of Dy-547-labeled cyclic diguanylate monophosphate (cdGMP) (red) and AlexaFluor-488 labeled recombinant MERS-CoV RBD antigen (green) in JAWS II cells following 24 h of incubation with RBD-NP (cdGMP). Abbreviations: Middle East respiratory syndrome-coronavirus receptor-binding domain (RBD) nanoparticles, MERS-CoV RBD-FR NPs; poly(lactic-*co*-glycolic acid), PLGA; nanoparticles, NPs; tobacco etch virus, TEV; cyclic diguanylate monophosphate, cdGMP. Reproduced with permission from ref (245). Copyright 2019 Wiley.

Ribonucleic acid conjugated to ferritin-based NPs have been proposed as a potent molecular chaperone.^243^ The use of this NP-based vaccine against MERS-CoV induced CD4^+^ T cells and promoted the production of TNF-α and IFN-γ (Figure 10). In another study, an immunogenic vaccine against MERS-CoV was introduced using a heterologous prime-boost method.^244^ Using a recombinant adenovirus serotype 5 that encodes the MERS-CoV spike gene (Ad5/MERS) and spike protein NPs, female BALB/c mice was immunized three times with the prime-boost vaccination. The homologous immunization with spike protein NPs successfully induced higher antibody titers. However, Th1 immune response was not generated by spike protein NPs. Only the Th2 immune response was elicited with the induction of neutralizing antibodies. A heterologous one-stage Ad5/MERS prime and two-stage spike protein NP boost appear to be more effective than the homologous prime-boost regimen in providing more durable immunogenicity and balance of Th1/Th2 responses.^244^

## 7 Translating Research into Clinical Practice

Translating archived knowledge acquired from the laboratory into clinical trials is a crucial and challenging stage for safe and tangible combat against COVID-19.^246^ To date, developing anti-COVID-19 drugs have encountered challenges because of their side effects to the lung and heart. A smart technology is therefore required for the design and fabrication of rational drugs that only target SARS-CoV-2 with minimal side effects.^247^ Drug repurposing is an effective drug discovery strategy based on the use of existing drugs. Such a strategy shortens the time and reduces the cost compared to de novo drug discovery. *In silico* pharmacology performed on a computer or via computer simulation is a smart, revolutionary technology for evaluating approved medicine, reducing the regulatory costs of innovation and decreasing the time for marketing of biomedical products. Such a “virtual” process is indispensable in contemporary drug discovery research for translating drugs into clinical trials.^248,249^ The combination of in silico strategy and large drug-related databases facilities the selection of appropriate repurposed drugs by screening their side effects on different organs. Drug-repurposing strategies have recently been performed by computational modeling on the interaction and mechanism of potential drugs with the host cells and SARS-CoV-2^250^ Computer modeling offers a platform for visual assessment and analysis of the molecular mechanisms involved in the entrance, replication, and transcription of virus molecules as well as their interactions with host cells, immune response, and the potential mechanisms of cell recovery.

Another research group investigated a deep-learning Dense Fully Convolutional Neural Network (DFCNN) model for screening established drugs against SARS-CoV-2 infection.^251^ In this approach, RNA sequences were collected from the Global Initiative on Sharing All Influenza Data (GISAID) database to investigate the 3D protein sequences and protein–ligand interactions via homology modeling. Drug screening was performed without using docking or molecular dynamics. This modeling successfully recognized chemical ligands (meglumine, vidarabine, adenosine, d-sorbitol, d-mannitol, sodium gluconate, ganciclovir, and chlorobutanol) and peptide drugs (combination of isoleucine, lysine, and proline) from the databases to aid scientists in identifying molecules that can combat SARS-CoV-2 in a shorter time period.

**Figure 11:**
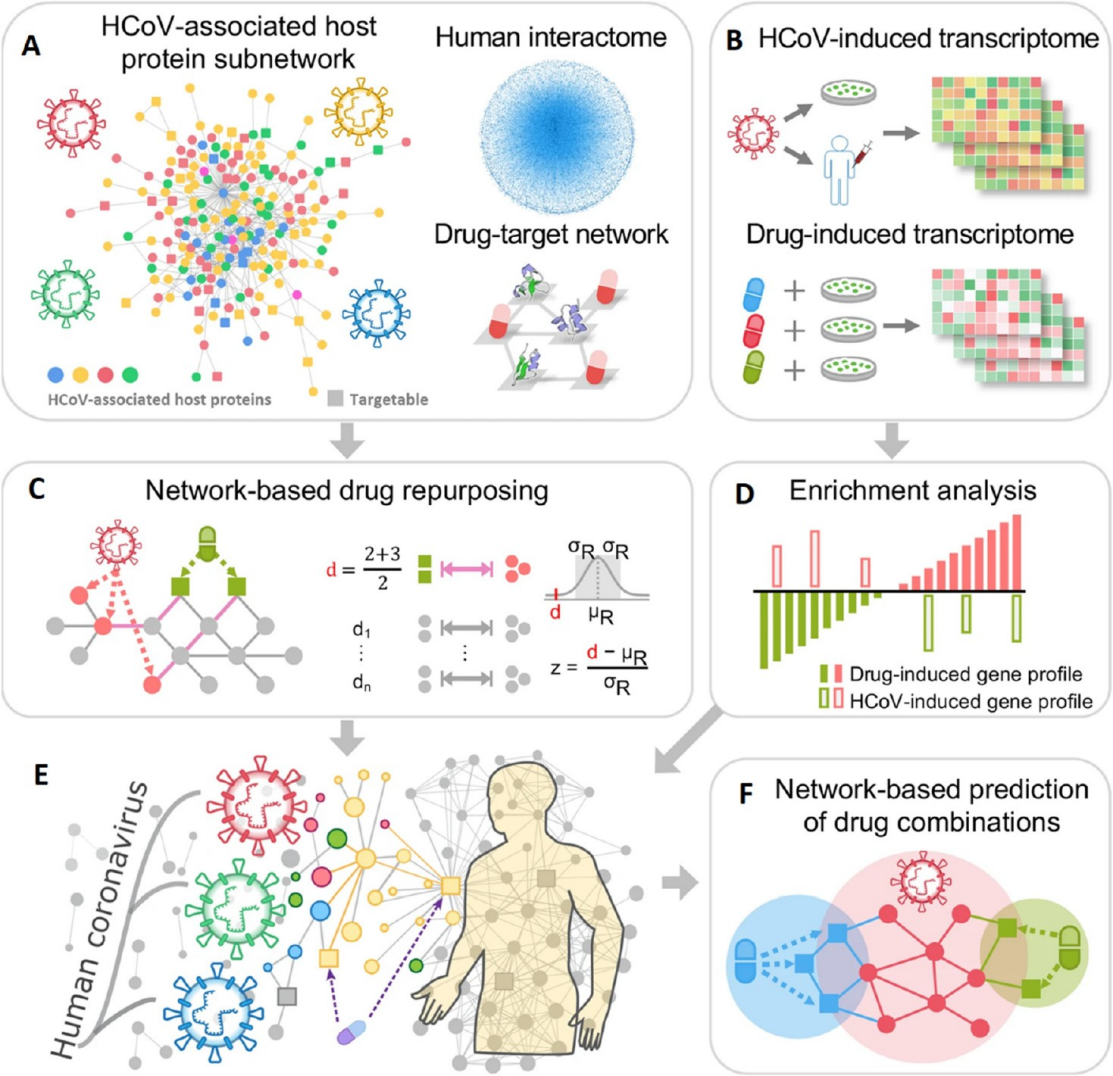
Network-based methodology constructed on a protein–protein network. (A) Human coronavirus (HCoV)-associated host proteins collected from the literature are pooled to generate a pan-HCoV protein subnetwork. (B) Screening of potential repurposable drug candidates via analyzing the network proximity between targeted drugs and proteins associated with HCoV. (C, D) Validation of network-based predictions using gene set enrichment analysis. (E) Network-based prediction of optimized drug combination using complementary exposure pattern. (F) Hypothetical illustration of the network-based methodology to explore protein–protein interactions constructed on the human interactome. Abbreviations: human coronavirus (HCoV). Reproduced with permission from ref (252). Copyright 2020 Springer Nature.

In another interesting work, an advanced pharmacology network-based approach was developed to evaluate a rational drug for effective treatment of COVID-19 infection (Figure 11).^252^ In this work, phylogenetic analysis of 15 human CoV whole genomes with coronavirus infection was performed. Using network proximity analyses of drug targets and human CoV–host interactions in the human interactome,^16^ potential anti-CoV repurposable drug candidates such as melatonin, mercaptopurine, and sirolimus were identified and further validated by enrichment analyses of drug–gene signatures in human cell lines. In addition, three potential drug combinations were identified through a “Complementary Exposure” pattern, including (i) sirolimus plus dactinomycin, (ii) mercaptopurine plus melatonin, and (iii) toremifene plus emodin. The study provides an excellent role model for the rapid identification of therapeutic drugs for combating SARS-CoV-2 infections.

Combining data generated on the mechanisms of COVID-19 infections with in silico models enables virologists, immunologists, clinicians, and computational biologists to collaborate in understanding the accurate molecular mechanisms of SARS-CoV-2 infection. This approach provides a useful guide for the development of advanced and efficient nanomedicine against the COVID-19 pandemic.

## 8 Challenges and Future Perspectives

Nanotechnology is rapidly becoming a vivid player in antiviral therapy for combating coronaviruses. Nanomaterials have been developed specifically to improve the delivery of biotherapeutics across physiological barriers, thereby resolving the classical challenge of low bioavailability.^253^ Nanomaterials possess various physicochemical and biological benefits. These benefits include reduced particle sizes that facilitate delivery through natural barriers, larger surface areas for higher drug loading, adjustable surface charge to facilitate drug entry across charged cell membranes, capability to anchor to targeting ligands to increase the specificity of the destined target, superior solubility and pharmacokinetic properties that result in longer circulation times, better accumulation, controlled/sustained release, and improved efficacy caused by either entrapping drug agents and protecting them from the physiological environment or surface modifications for targeting purposes.^254−256^

The application of nanomaterials as drug carriers, however, is not free from challenges. One of the most eminent challenge is their degradation prior to reaching the target. Nanoparticles, for example, are degraded in the gastrointestinal tract when they are administrated orally. Nanoparticles are not always successful in crossing the mucus barrier, which results in reduced or nonabsorption.^257^ Other challenges associated with the use of nanomaterials include interactions with biological molecules that result in opsonization, phagocytosis by macrophages that reduces their plasma half-life,^258^ nonspecific absorption which induces apoptosis of the cells that absorb them, and disruption of their cell membranes.^259^

An ideal nanocarrier for proficient antiviral treatment needs to possess several attributes. These attributes include: (1) excellent clinical outcome, as therapeutic devices are required to be effective, available, targeted, safe, and affordable; (2) the nanocarrier needs to improve the efficacy of drug delivery, reduce intake rate and time, decrease side effects, and reduce the cost of therapy; (3) the nanocarrier should possess an appropriate fabrication design that permits targeted drug delivery in a sustained released manner. Hybrid nanosystems have the potential to meet the requirements for nanomanufacturing and shape/size configurations. In addition, the nanomaterials used for fabricating the designated compositions should be biodegradable, biocompatible, and nontoxic. In this regard, polymers offer tremendous potential for chemical surface modifications. More complex challenges are associated with nanocarrier shape because this property is associated with NP size and surface charge. Polymer-based nanomaterials such as polyethylene glycol and poly(lactide-*co*-glycolide) are close-to-ideal candidates because of their flexibility to uptake various charges, capacity to be fabricated in different shapes and sizes for enhancing the permissibility of the composition, and reduced clearance to prolong circulation time.^260^ Polymeric nanomaterials are likely to emerge as the materials of choice for the development of vaccine and drug carriers for single-dose and needle-free delivery.^261^

**Figure 12:**
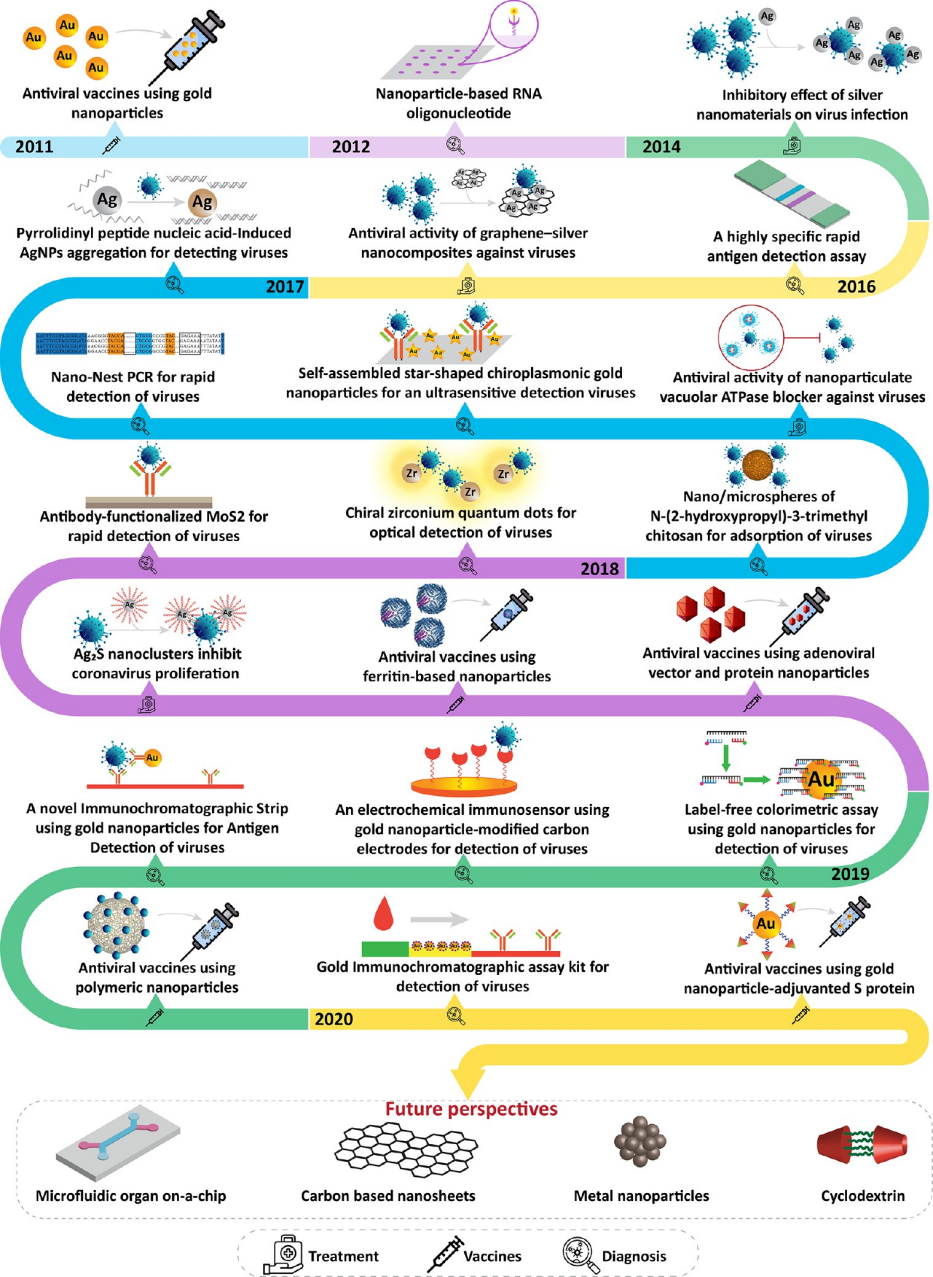
Potential use of nanobiotechnology for biosensing, nanomedicine, and nanovaccine components against coronaviruses.

Metal NPs such as AgNPs, AuNPs, MNPs, and their related compositions may be used as alternative candidates for the delivery of therapeutic agents against CoVs.^262^ Because the size of the devices influences their biodistribution and rate of uptake, a nanocarrier has to be used in the nanometer size range (e.g., <200 nm).^263^ For cyclodextrin drug delivery systems such as hydroxypropyl beta-cyclodextrins (HPβCD), the use of carbon-based nanosheets may overcome formulation challenges of antiviral drugs by improving solubility and bioavailability.^264,265^ Likewise, they may be used as safe and efficient adjuvants in vaccines for coronaviruses. Figure 12 summarizes the trend of nanobiotechnology against CoVs.

**Figure 13:**
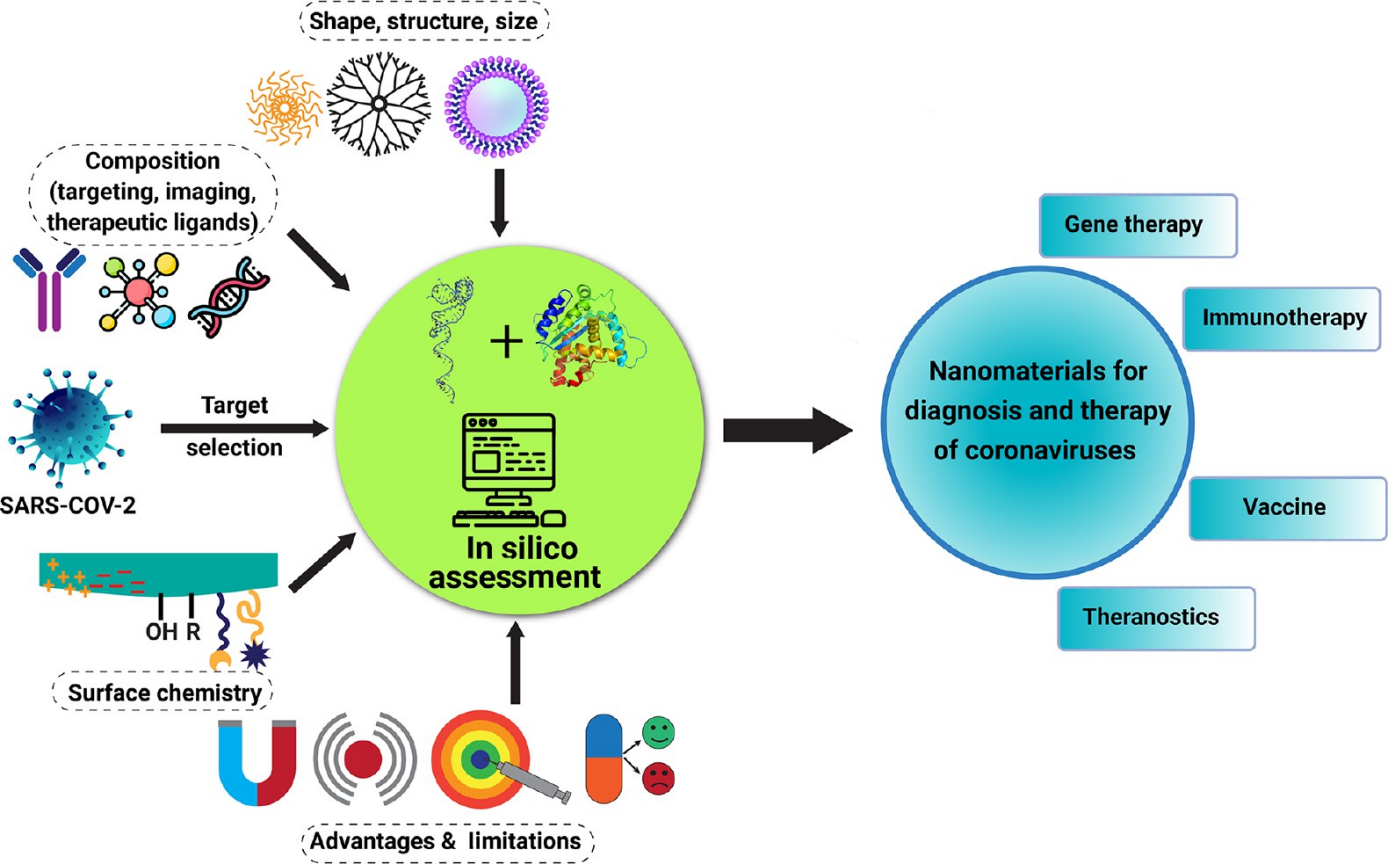
Rational design of nanomaterials using in silico assessments for effective diagnosis and treatment of CoV infections.

In the grand scheme of things, the applications of nanoplatforms for the detection of human coronaviruses have yet remained unresolved for nanotechnology researchers. Colorimetric sensing, electrochemiluminescence, immunosensing, photoluminescence, and chiroimmunosensing, as well as electrochemical sensors, are potential techniques to detect coronaviruses. Various nanobased vaccines have demonstrated the potential to induce a more potent immune response. However, further investigations on the interaction of virus particles with host cells are required to tackle the application of smart NPs against the mutated versions of highly contagious SARS-CoV2 (Figure 13).

As of February 2021, eight COVID-19 vaccines based on different technologies have been approved or authorized for emergency use. They are the mRNA vaccines BNT162 from Pfizer/BioNtech and mRNA-1273 from Moderna, the chimpanzee adenovirus-based AZD1222 (Covidshield) vaccine from Oxford-Astra Zeneca, the Ad26-based viral vector vaccine from Johnson &Johnson, the virus-inactivated Covaxin vaccine from Indian Barhat Biotech, the CoronaVac vaccine from Sinovac Biotech, China, and the human adenovirus-based Sputnik V vaccine from the Gamaleya National Center of Epidemiology and Microbiology, Russia.^266,267^ Although more than 250 other vaccines are in various stages of development, the emergence of new SARS-CoV2 variants^268^ with possible highly transmissibility demonstrate the urgency of developing new vaccine formulations with high effectiveness.

## References

[ref1] Guan W.-j.; Ni Z.-y.; Hu Y.; Liang W.-h.; Ou C.-q.; He J.-x.; Liu L.; Shan H.; Lei C.-l.; Hui D. S. Clinical characteristics of 2019 novel coronavirus infection in China. MedRxiv,2020, 10.1101/2020.02.06.20020974.

[ref2] Sohrabi C.; Alsafi Z.; O’Neill N.; Khan M.; Kerwan A.; Al-Jabir A.; Iosifidis C.; Agha R. World Health Organization declares global emergency: A review of the 2019 novel coronavirus (COVID-19). International Journal of Surgery 2020, 76 (76), 71–76. 10.1016/j.ijsu.2020.02.034. 32112977 PMC7105032

[ref3] Bosch B. J.; Martina B. E.; van der Zee R.; Lepault J.; Haijema B. J.; Versluis C.; Heck A. J.; de Groot R.; Osterhaus A. D.; Rottier P. J. Severe acute respiratory syndrome coronavirus (SARS-CoV) infection inhibition using spike protein heptad repeat-derived peptides. Proc. Natl. Acad. Sci. U. S. A. 2004, 101 (22), 8455–8460. 10.1073/pnas.0400576101. 15150417 PMC420415

[ref4] World Health Organiation. Middle East Respiratory Syndrome Coronavirus (MERS-cov). http://www.who.int/emergencies/mers-cov/en (accessed 2021-01).

[ref5] Huang C.; Wang Y.; Li X.; Ren L.; Zhao J.; Hu Y.; Zhang L.; Fan G.; Xu J.; Gu X. Clinical features of patients infected with 2019 novel coronavirus in Wuhan, China. Lancet 2020, 395 (10223), 497–506. 10.1016/S0140-6736(20)30183-5. 31986264 PMC7159299

[ref6] Holshue M. L.; DeBolt C.; Lindquist S.; Lofy K. H.; Wiesman J.; Bruce H.; Spitters C.; Ericson K.; Wilkerson S.; Tural A.; et al. First case of 2019 novel coronavirus in the United States. N. Engl. J. Med. 2020, 382, 929 10.1056/NEJMoa2001191. 32004427 PMC7092802

[ref7] World Health Organization. https://covid19.who.int/ (accessed 2021-02).

[ref8] Kargozar S.; Mozafari M. Nanotechnology and Nanomedicine: Start small, think big. Materials Today: Proceedings 2018, 5 (7), 15492–15500. 10.1016/j.matpr.2018.04.155.

[ref9] Yang Y.; Chawla A.; Zhang J.; Esa A.; Jang H. L.; Khademhosseini A., Applications of nanotechnology for regenerative medicine; healing tissues at the nanoscale. In Principles of Regenerative Medicine; Elsevier, 2019; pp 485–504.

[ref10] Lin L. C. W.; Chattopadhyay S.; Lin J. C.; Hu C. M. J. Advances and opportunities in nanoparticle-and nanomaterial-based vaccines against bacterial infections. Adv. Healthcare Mater. 2018, 7 (13), 1701395 10.1002/adhm.201701395. 29508547

[ref11] Cojocaru F.-D.; Botezat D.; Gardikiotis I.; Uritu C.-M.; Dodi G.; Trandafir L.; Rezus C.; Rezus E.; Tamba B.-I.; Mihai C.-T. Nanomaterials designed for antiviral drug delivery transport across biological barriers. Pharmaceutics 2020, 12 (2), 171 10.3390/pharmaceutics12020171. PMC7076512 32085535

[ref12] Nasrollahzadeh M.; Sajjadi M.; Soufi G. J.; Iravani S.; Varma R. S. Nanomaterials and Nanotechnology-Associated Innovations against Viral Infections with a Focus on Coronaviruses. Nanomaterials 2020, 10 (6), 1072 10.3390/nano10061072. PMC7352498 32486364

[ref13] Ksiazek T. G.; Erdman D.; Goldsmith C. S.; Zaki S. R.; Peret T.; Emery S.; Tong S.; Urbani C.; Comer J. A.; Lim W.; et al. A novel coronavirus associated with severe acute respiratory syndrome. N. Engl. J. Med. 2003, 348 (20), 1953–1966. 10.1056/NEJMoa030781. 12690092

[ref14] Kuiken T.; Fouchier R. A.; Schutten M.; Rimmelzwaan G. F.; Van Amerongen G.; van Riel D.; Laman J. D.; de Jong T.; van Doornum G.; Lim W.; et al. Newly discovered coronavirus as the primary cause of severe acute respiratory syndrome. Lancet 2003, 362 (9380), 263–270. 10.1016/S0140-6736(03)13967-0. 12892955 PMC7112434

[ref15] Villamil-Gómez W. E.; Sánchez Á.; Gelis L.; Silvera L. A.; Barbosa J.; Otero-Nader O.; Bonilla-Salgado C. D.; Rodríguez-Morales A. J. Fatal human coronavirus 229E (HCoV-229E) and RSV–Related pneumonia in an AIDS patient from Colombia. Travel medicine and infectious disease 2020, 36, 101573 10.1016/j.tmaid.2020.101573. 32035270 PMC7128323

[ref16] Oong X. Y.; Ng K. T.; Takebe Y.; Ng L. J.; Chan K. G.; Chook J. B.; Kamarulzaman A.; Tee K. K. Identification and evolutionary dynamics of two novel human coronavirus OC43 genotypes associated with acute respiratory infections: phylogenetic, spatiotemporal and transmission network analyses. Emerging Microbes Infect. 2017, 6 (1), 1–13. 10.1038/emi.2016.132. PMC5285497 28050020

[ref17] Friedman N.; Alter H.; Hindiyeh M.; Mendelson E.; Shemer Avni Y.; Mandelboim M. Human coronavirus infections in Israel: epidemiology, clinical symptoms and summer seasonality of HCoV-HKU1. Viruses 2018, 10 (10), 515 10.3390/v10100515. PMC6213580 30241410

[ref18] de Groot R. J.; Baker S. C.; Baric R. S.; Brown C. S.; Drosten C.; Enjuanes L.; Fouchier R. A.; Galiano M.; Gorbalenya A. E.; Memish Z. A.; et al. Middle East respiratory syndrome coronavirus (MERS-CoV): announcement of the Coronavirus Study Group. J. Virol. 2013, 87 (14), 7790–7792. 10.1128/JVI.01244-13. 23678167 PMC3700179

[ref19] Lai C.-C.; Shih T.-P.; Ko W.-C.; Tang H.-J.; Hsueh P.-R. Severe acute respiratory syndrome coronavirus 2 (SARS-CoV-2) and corona virus disease-2019 (COVID-19): the epidemic and the challenges. Int. J. Antimicrob. Agents 2020, 55, 105924 10.1016/j.ijantimicag.2020.105924. 32081636 PMC7127800

[ref20] Enjuanes Sánchez L.; Zúñiga Lucas S.; Castaño-Rodríguez C.; Gutierrez-Alvarez J.; Cantón J.; Solá Gurpegui I., Molecular basis of Coronavirus virulence and vaccine development. Advances in Virus Research; Elsevier, 2016; Vol. 96, pp 245–286. 27712626 10.1016/bs.aivir.2016.08.003 PMC7112271

[ref21] Mousavizadeh L.; Ghasemi S. Genotype and phenotype of COVID-19: Their roles in pathogenesis. Journal of Microbiology, Immunology and Infection 2021, 54, 159 10.1016/j.jmii.2020.03.022. PMC7138183 32265180

[ref22] Hoffmann M.; Kleine-Weber H.; Krüger N.; Mueller M. A.; Drosten C.; Pöhlmann S. The novel coronavirus 2019 (2019-nCoV) uses the SARS-coronavirus receptor ACE2 and the cellular protease TMPRSS2 for entry into target cells. BioRxiv, 2020, 10.1016/j.jmii.2020.03.022.

[ref23] Nakagawa K.; Makino S. Mechanisms of Coronavirus Nsp1-Mediated Control of Host and Viral Gene Expression. Cells 2021, 10 (2), 300 10.3390/cells10020300. 33540583 PMC7912902

[ref24] Woo P. C.; Huang Y.; Lau S. K.; Yuen K.-Y. Coronavirus genomics and bioinformatics analysis. Viruses 2010, 2 (8), 1804–1820. 10.3390/v2081803. 21994708 PMC3185738

[ref25] Lim Y. X.; Ng Y. L.; Tam J. P.; Liu D. X. Human coronaviruses: a review of virus–host interactions. Diseases 2016, 4 (3), 26 10.3390/diseases4030026. PMC5456285 28933406

[ref26] Yang D.; Leibowitz J. L. The structure and functions of coronavirus genomic 3′ and 5′ ends. Virus Res. 2015, 206, 120–133. 10.1016/j.virusres.2015.02.025. 25736566 PMC4476908

[ref27] van Boheemen S.; de Graaf M.; Lauber C.; Bestebroer T. M.; Raj V. S.; Zaki A. M.; Osterhaus A. D. M. E.; Haagmans B. L.; Gorbalenya A. E.; Snijder E. J.; Fouchier R. A. M. Genomic characterization of a newly discovered coronavirus associated with acute respiratory distress syndrome in humans. mBio 2012, 3 (6), e00473 10.1128/mBio.00473-12. 23170002 PMC3509437

[ref28] Liu D. X.; Fung T. S.; Chong K. K.-L.; Shukla A.; Hilgenfeld R. Accessory proteins of SARS-CoV and other coronaviruses. Antiviral Res. 2014, 109, 97–109. 10.1016/j.antiviral.2014.06.013. 24995382 PMC7113789

[ref29] Chen Y.; Liu Q.; Guo D. Emerging coronaviruses: genome structure, replication, and pathogenesis. J. Med. Virol. 2020, 92 (4), 418–423. 10.1002/jmv.25681. 31967327 PMC7167049

[ref30] Neuman B. W.; Kiss G.; Kunding A. H.; Bhella D.; Baksh M. F.; Connelly S.; Droese B.; Klaus J. P.; Makino S.; Sawicki S. G.; et al. A structural analysis of M protein in coronavirus assembly and morphology. J. Struct. Biol. 2011, 174 (1), 11–22. 10.1016/j.jsb.2010.11.021. 21130884 PMC4486061

[ref31] Benvenuto D.; Giovanetti M.; Ciccozzi A.; Spoto S.; Angeletti S.; Ciccozzi M. The 2019-new coronavirus epidemic: evidence for virus evolution. J. Med. Virol. 2020, 92 (4), 455–459. 10.1002/jmv.25688. 31994738 PMC7166400

[ref32] Lu R.; Zhao X.; Li J.; Niu P.; Yang B.; Wu H.; Wang W.; Song H.; Huang B.; Zhu N.; et al. Genomic characterisation and epidemiology of 2019 novel coronavirus: implications for virus origins and receptor binding. Lancet 2020, 395 (10224), 565–574. 10.1016/S0140-6736(20)30251-8. 32007145 PMC7159086

[ref33] Li X.; Luk H. K.; Lau S. K.; Woo P. C. Human Coronaviruses: General Features. Reference Module in Biomedical Sciences 2019, B978-0-12-801238-3.95704-0 10.1016/B978-0-12-801238-3.95704-0.

[ref34] Walsh E. E.; Shin J. H.; Falsey A. R. Clinical impact of human coronaviruses 229E and OC43 infection in diverse adult populations. J. Infect. Dis. 2013, 208 (10), 1634–1642. 10.1093/infdis/jit393. 23922367 PMC3805243

[ref35] McBride R.; Fielding B. C. The role of severe acute respiratory syndrome (SARS)-coronavirus accessory proteins in virus pathogenesis. Viruses 2012, 4 (11), 2902–2923. 10.3390/v4112902. 23202509 PMC3509677

[ref36] Pene F.; Merlat A.; Vabret A.; Rozenberg F.; Buzyn A.; Dreyfus F.; Cariou A.; Freymuth F.; Lebon P. Coronavirus 229E-related pneumonia in immunocompromised patients. Clin. Infect. Dis. 2003, 37 (7), 929–932. 10.1086/377612. 13130404 PMC7107892

[ref37] Jacomy H.; Fragoso G.; Almazan G.; Mushynski W. E.; Talbot P. J. Human coronavirus OC43 infection induces chronic encephalitis leading to disabilities in BALB/C mice. Virology 2006, 349 (2), 335–346. 10.1016/j.virol.2006.01.049. 16527322 PMC7111850

[ref38] Jones B. A.; Grace D.; Kock R.; Alonso S.; Rushton J.; Said M. Y.; McKeever D.; Mutua F.; Young J.; McDermott J.; Pfeiffer D. U. Zoonosis emergence linked to agricultural intensification and environmental change. Proc. Natl. Acad. Sci. U. S. A. 2013, 110 (21), 8399–8404. 10.1073/pnas.1208059110. 23671097 PMC3666729

[ref39] Bertram S.; Dijkman R.; Habjan M.; Heurich A.; Gierer S.; Glowacka I.; Welsch K.; Winkler M.; Schneider H.; Hofmann-Winkler H.; et al. TMPRSS2 activates the human coronavirus 229E for cathepsin-independent host cell entry and is expressed in viral target cells in the respiratory epithelium. Journal of virology 2013, 87 (11), 6150–6160. 10.1128/JVI.03372-12. 23536651 PMC3648130

[ref40] Bosch B. J.; Bartelink W.; Rottier P. J. Cathepsin L functionally cleaves the SARS-CoV class I fusion protein upstream of rather than adjacent to the fusion peptide. J. Virol. 2008, 82, 8887 10.1128/JVI.00415-08. 18562523 PMC2519682

[ref41] Qi F.; Qian S.; Zhang S.; Zhang Z. Single cell RNA sequencing of 13 human tissues identify cell types and receptors of human coronaviruses. Biochem. Biophys. Res. Commun. 2020, 526 (1), 135–140. 10.1016/j.bbrc.2020.03.044. 32199615 PMC7156119

[ref42] Sola I.; Almazan F.; Zuniga S.; Enjuanes L. Continuous and discontinuous RNA synthesis in coronaviruses. Annu. Rev. Virol. 2015, 2, 265–288. 10.1146/annurev-virology-100114-055218. 26958916 PMC6025776

[ref43] Xu J.; Zhao S.; Teng T.; Abdalla A. E.; Zhu W.; Xie L.; Wang Y.; Guo X. Systematic comparison of two animal-to-human transmitted human coronaviruses: SARS-CoV-2 and SARS-CoV. Viruses 2020, 12 (2), 244 10.3390/v12020244. PMC7077191 32098422

[ref44] Glebov O. O. Understanding SARS-CoV-2 endocytosis for COVID-19 drug repurposing. FEBS J. 2020, 287, 3664 10.1111/febs.15369. 32428379 PMC7276759

[ref45] Pelkmans L.; Helenius A. Insider information: what viruses tell us about endocytosis. Curr. Opin. Cell Biol. 2003, 15 (4), 414–422. 10.1016/S0955-0674(03)00081-4. 12892781

[ref46] Walls A. C.; Park Y.-J.; Tortorici M. A.; Wall A.; McGuire A. T.; Veesler D. Structure, function, and antigenicity of the SARS-CoV-2 spike glycoprotein. Cell 2020, 181 (2), 281–292. 10.1016/j.cell.2020.02.058. 32155444 PMC7102599

[ref47] Shang J.; Wan Y.; Luo C.; Ye G.; Geng Q.; Auerbach A.; Li F. Cell entry mechanisms of SARS-CoV-2. Proc. Natl. Acad. Sci. U. S. A. 2020, 117 (21), 11727–11734. 10.1073/pnas.2003138117. 32376634 PMC7260975

[ref48] Cantuti-Castelvetri L.; Ojha R.; Pedro L. D.; Djannatian M.; Franz J.; Kuivanen S.; van der Meer F.; Kallio K.; Kaya T.; Anastasina M.; et al. Neuropilin-1 facilitates SARS-CoV-2 cell entry and infectivity. Science 2020, 370 (6518), 856–860. 10.1126/science.abd2985. 33082293 PMC7857391

[ref49] Naicker S.; Yang C.-W.; Hwang S.-J.; Liu B.-C.; Chen J.-H.; Jha V. The Novel Coronavirus 2019 epidemic and kidneys. Kidney Int. 2020, 97 (5), 824–828. 10.1016/j.kint.2020.03.001. 32204907 PMC7133222

[ref50] Wang Q.; Zhang Y.; Wu L.; Niu S.; Song C.; Zhang Z.; Lu G.; Qiao C.; Hu Y.; Yuen K.-Y.; et al. Structural and functional basis of SARS-CoV-2 entry by using human ACE2. Cell 2020, 181 (4), 894–904. 10.1016/j.cell.2020.03.045. 32275855 PMC7144619

[ref51] Tang T.; Bidon M.; Jaimes J. A.; Whittaker G. R.; Daniel S. Coronavirus membrane fusion mechanism offers a potential target for antiviral development. Antiviral Res. 2020, 178, 104792 10.1016/j.antiviral.2020.104792. 32272173 PMC7194977

[ref52] Bayati A.; Kumar R.; Francis V.; McPherson P. S. SARS-CoV-2 infects cells following viral entry via clathrin-mediated endocytosis. J. Biol. Chem. 2021, 296, 100306 10.1016/j.jbc.2021.100306. 33476648 PMC7816624

[ref53] Wang H.; Yang P.; Liu K.; Guo F.; Zhang Y.; Zhang G.; Jiang C. SARS coronavirus entry into host cells through a novel clathrin-and caveolae-independent endocytic pathway. Cell Res. 2008, 18 (2), 290–301. 10.1038/cr.2008.15. 18227861 PMC7091891

[ref54] Tan Y.-J.; Teng E.; Shen S.; Tan T. H.; Goh P.-Y.; Fielding B. C.; Ooi E.-E.; Tan H.-C.; Lim S. G.; Hong W. A novel severe acute respiratory syndrome coronavirus protein, U274, is transported to the cell surface and undergoes endocytosis. J. Virol. 2004, 78 (13), 6723–6734. 10.1128/JVI.78.13.6723-6734.2004. 15194747 PMC421683

[ref55] Takano T.; Wakayama Y.; Doki T. Endocytic pathway of feline coronavirus for cell entry: differences in serotype-dependent viral entry pathway. Pathogens 2019, 8 (4), 300 10.3390/pathogens8040300. PMC6963708 31888266

[ref56] Hartenian E.; Nandakumar D.; Lari A.; Ly M.; Tucker J. M.; Glaunsinger B. A. The molecular virology of Coronaviruses. J. Biol. Chem. 2020, 295 (37), 12910–12934. 10.1074/jbc.REV120.013930. 32661197 PMC7489918

[ref57] Yang N.; Shen H.-M. Targeting the endocytic pathway and autophagy process as a novel therapeutic strategy in COVID-19. Int. J. Biol. Sci. 2020, 16 (10), 1724 10.7150/ijbs.45498. 32226290 PMC7098027

[ref58] Li G.; Fan Y.; Lai Y.; Han T.; Li Z.; Zhou P.; Pan P.; Wang W.; Hu D.; Liu X.; et al. Coronavirus infections and immune responses. J. Med. Virol. 2020, 92 (4), 424–432. 10.1002/jmv.25685. 31981224 PMC7166547

[ref59] Xiao F.; Tang M.; Zheng X.; Liu Y.; Li X.; Shan H. Evidence for gastrointestinal infection of SARS-CoV-2. Gastroenterology 2020, 158 (6), 1831–1833. 10.1053/j.gastro.2020.02.055. 32142773 PMC7130181

[ref60] Arbour N.; Day R.; Newcombe J.; Talbot P. J. Neuroinvasion by human respiratory coronaviruses. J. Virol. 2000, 74 (19), 8913–8921. 10.1128/JVI.74.19.8913-8921.2000. 10982334 PMC102086

[ref61] Gavriatopoulou M.; Korompoki E.; Fotiou D.; Ntanasis-Stathopoulos I.; Psaltopoulou T.; Kastritis E.; Terpos E.; Dimopoulos M. A. Organ-specific manifestations of COVID-19 infection. Clin. Exp. Med. 2020, 20 (4), 493–506. 10.1007/s10238-020-00648-x. 32720223 PMC7383117

[ref62] ME O. B.; Thurman A.; Pezzulo A.; Leidinger M.; Klesney-Tait J.; Karp P.; Tan P.; Wohlford-Lenane C.; McCray Jr P.; Meyerholz D. Heterogeneous expression of the SARS-Coronavirus-2 receptor ACE2 in the human respiratory tract. Biorxiv, 2020, 10.1101/2020.04.22.056127. PMC7505653 32971472

[ref63] Gavriatopoulou M.; Korompoki E.; Fotiou D.; Ntanasis-Stathopoulos I.; Psaltopoulou T.; Kastritis E.; Terpos E.; Dimopoulos M. A. Organ-specific manifestations of COVID-19 infection. Clin. Exp. Med. 2020, 20, 493 10.1007/s10238-020-00648-x. 32720223 PMC7383117

[ref64] McGonagle D.; O’Donnell J. S.; Sharif K.; Emery P.; Bridgewood C. Immune mechanisms of pulmonary intravascular coagulopathy in COVID-19 pneumonia. Lancet Rheumatology 2020, 2, e437 10.1016/S2665-9913(20)30121-1. 32835247 PMC7252093

[ref65] Numbers K.; Brodaty H. The effects of the COVID-19 pandemic on people with dementia. Nat. Rev. Neurol. 2021, 17, 69 10.1038/s41582-020-00450-z. 33408384 PMC7786184

[ref66] Suhail S.; Zajac J.; Fossum C.; Lowater H.; McCracken C.; Severson N.; Laatsch B.; Narkiewicz-Jodko A.; Johnson B.; Liebau J.; et al. Role of Oxidative Stress on SARS-CoV (SARS) and SARS-CoV-2 (COVID-19) Infection: A Review. Protein J. 2020, 39, 644–656. 10.1007/s10930-020-09935-8. 33106987 PMC7587547

[ref67] Atal S.; Fatima Z. IL-6 inhibitors in the treatment of serious COVID-19: a promising therapy?. Pharm. Med. 2020, 34 (4), 223–231. 10.1007/s40290-020-00342-z. PMC7292936 32535732

[ref68] Horby P.; Lim W. S.; Emberson J.; Mafham M.; Bell J.; Linsell L.; Staplin N.; Brightling C.; Ustianowski A.; Elmahi E. Dexamethasone in hospitalized patients with Covid-19. N. Engl. J. Med. 2021, 384, 693–704. 10.1056/NEJMoa2021436. 32678530 PMC7383595

[ref69] Singh A. K.; Bhushan B.; Maurya A.; Mishra G.; Singh S. K.; Awasthi R. Novel Coronavirus disease 2019 (COVID-19) and neurodegenerative disorders. Dermatologic Therapy 2020, 33, e13591 10.1111/dth.13591. 32412679 PMC7261984

[ref70] Gomez-Pinedo U.; Matias-Guiu J.; Sanclemente-Alaman I.; Moreno-Jimenez L.; Montero-Escribano P.; Matias-Guiu J. A. SARS-CoV2 as a potential trigger of neurodegenerative diseases. Mov. Disord. 2020, 35, 1104 10.1002/mds.28179. 32502296 PMC7300724

[ref71] Favreau D. J.; Meessen-Pinard M.; Desforges M.; Talbot P. J. Human coronavirus-induced neuronal programmed cell death is cyclophilin d dependent and potentially caspase dispensable. Journal of virology 2012, 86 (1), 81–93. 10.1128/JVI.06062-11. 22013052 PMC3255912

[ref72] Desforges M.; Le Coupanec A.; Brison É.; Meessen-Pinard M.; Talbot P. J., Neuroinvasive and neurotropic human respiratory coronaviruses: potential neurovirulent agents in humans. In Infectious Diseases and Nanomedicine I; Springer, 2014; pp 75–96. 10.1007/978-81-322-1777-0_6 PMC7121612 24619619

[ref73] De Felice F. G.; Tovar-Moll F.; Moll J.; Munoz D. P.; Ferreira S. T. Severe Acute Respiratory Syndrome Coronavirus 2 (SARS-CoV-2) and the Central Nervous System. Trends Neurosci. 2020, 43, 355–357. 10.1016/j.tins.2020.04.004. 32359765 PMC7172664

[ref74] Mao L.; Jin H.; Wang M.; Hu Y.; Chen S.; He Q.; Chang J.; Hong C.; Zhou Y.; Wang D.; et al. Neurologic manifestations of hospitalized patients with coronavirus disease 2019 in Wuhan, China. JAMA Neurol. 2020, 77 (6), 683–690. 10.1001/jamaneurol.2020.1127. 32275288 PMC7149362

[ref75] Helms J.; Kremer S.; Merdji H.; Clere-Jehl R.; Schenck M.; Kummerlen C.; Collange O.; Boulay C.; Fafi-Kremer S.; Ohana M.; et al. Neurologic features in severe SARS-CoV-2 infection. N. Engl. J. Med. 2020, 382 (23), 2268–2270. 10.1056/NEJMc2008597. 32294339 PMC7179967

[ref76] Severance E. G.; Dickerson F. B.; Viscidi R. P.; Bossis I.; Stallings C. R.; Origoni A. E.; Sullens A.; Yolken R. H. Koroonaviirus COVID-19 ja skisofreenia. Schizophr Bull. 2011, 37 (1), 101–107. 10.1093/schbul/sbp052. 19491313 PMC3004184

[ref77] Chan J. F.-W.; Chan K.-H.; Choi G. K.-Y.; To K. K.-W.; Tse H.; Cai J.-P.; Yeung M. L.; Cheng V. C.-C.; Chen H.; Che X.-Y.; et al. Differential cell line susceptibility to the emerging novel human betacoronavirus 2c EMC/2012: implications for disease pathogenesis and clinical manifestation. J. Infect. Dis. 2013, 207 (11), 1743–1752. 10.1093/infdis/jit123. 23532101 PMC7107374

[ref78] Paniz-Mondolfi A.; Bryce C.; Grimes Z.; Gordon R. E.; Reidy J.; Lednicky J.; Sordillo E. M.; Fowkes M. Central nervous system involvement by severe acute respiratory syndrome coronavirus-2 (SARS-CoV-2). J. Med. Virol. 2020, 92 (7), 699–702. 10.1002/jmv.25915. 32314810 PMC7264598

[ref79] Hocke A. C.; Becher A.; Knepper J.; Peter A.; Holland G.; Tönnies M.; Bauer T. T.; Schneider P.; Neudecker J.; Muth D.; et al. Emerging human middle East respiratory syndrome coronavirus causes widespread infection and alveolar damage in human lungs. Am. J. Respir. Crit. Care Med. 2013, 188 (7), 882–886. 10.1164/rccm.201305-0954LE. 24083868

[ref80] Pillaiyar T.; Meenakshisundaram S.; Manickam M. Recent discovery and development of inhibitors targeting coronaviruses. Drug Discovery Today 2020, 25 (4), 668–688. 10.1016/j.drudis.2020.01.015. 32006468 PMC7102522

[ref81] Liu J.; Li S.; Liu J.; Liang B.; Wang X.; Wang H.; Li W.; Tong Q.; Yi J.; Zhao L.; et al. Longitudinal characteristics of lymphocyte responses and cytokine profiles in the peripheral blood of SARS-CoV-2 infected patients. EBioMedicine 2020, 55, 102763 10.1016/j.ebiom.2020.102763. 32361250 PMC7165294

[ref82] Jando J.; Camargo S. M.; Herzog B.; Verrey F. Expression and regulation of the neutral amino acid transporter B0AT1 in rat small intestine. PLoS One 2017, 12 (9), e0184845 10.1371/journal.pone.0184845. 28915252 PMC5600382

[ref83] Zheng Y.-Y.; Ma Y.-T.; Zhang J.-Y.; Xie X. COVID-19 and the cardiovascular system. Nat. Rev. Cardiol. 2020, 17 (5), 259–260. 10.1038/s41569-020-0360-5. 32139904 PMC7095524

[ref84] Alhogbani T. Acute myocarditis associated with novel Middle East respiratory syndrome coronavirus. Annals of Saudi medicine 2016, 36 (1), 78–80. 10.5144/0256-4947.2016.78. 26922692 PMC6074274

[ref85] Hoffmann M.; Kleine-Weber H.; Schroeder S.; Krüger N.; Herrler T.; Erichsen S.; Schiergens T. S.; Herrler G.; Wu N.-H.; Nitsche A.; et al. SARS-CoV-2 cell entry depends on ACE2 and TMPRSS2 and is blocked by a clinically proven protease inhibitor. Cell 2020, 181 (2), 271–280. 10.1016/j.cell.2020.02.052. 32142651 PMC7102627

[ref86] Clerkin K. J.; Fried J. A.; Raikhelkar J.; Sayer G.; Griffin J. M.; Masoumi A.; Jain S. S.; Burkhoff D.; Kumaraiah D.; Rabbani L.; et al. COVID-19 and cardiovascular disease. Circulation 2020, 141 (20), 1648–1655. 10.1161/CIRCULATIONAHA.120.046941. 32200663

[ref87] Feng G.; Zheng K. I.; Yan Q.-Q.; Rios R. S.; Targher G.; Byrne C. D.; Poucke S. V.; Liu W.-Y.; Zheng M.-H. COVID-19 and liver dysfunction: current insights and emergent therapeutic strategies. Journal of clinical and translational hepatology 2020, 8 (1), 1 10.14218/JCTH.2020.00018. 32274342 PMC7132016

[ref88] Farcas G. A.; Poutanen S. M.; Mazzulli T.; Willey B. M.; Butany J.; Asa S. L.; Faure P.; Akhavan P.; Low D. E.; Kain K. C. Fatal severe acute respiratory syndrome is associated with multiorgan involvement by coronavirus. J. Infect. Dis. 2005, 191 (2), 193–197. 10.1086/426870. 15609228 PMC7109982

[ref89] Li W.; Moore M. J.; Vasilieva N.; Sui J.; Wong S. K.; Berne M. A.; Somasundaran M.; Sullivan J. L.; Luzuriaga K.; Greenough T. C.; et al. Angiotensin-converting enzyme 2 is a functional receptor for the SARS coronavirus. Nature 2003, 426 (6965), 450–454. 10.1038/nature02145. 14647384 PMC7095016

[ref90] Chai X.; Hu L.; Zhang Y.; Han W.; Lu Z.; Ke A.; Zhou J.; Shi G.; Fang N.; Fan J. Specific ACE2 expression in cholangiocytes may cause liver damage after 2019-nCoV infection. biorxiv, 2020, 10.1101/2020.02.03.931766.

[ref91] Tang L. S.; Covert E.; Wilson E.; Kottilil S. Chronic hepatitis B infection: a review. Jama 2018, 319 (17), 1802–1813. 10.1001/jama.2018.3795. 29715359

[ref92] Saad M.; Omrani A. S.; Baig K.; Bahloul A.; Elzein F.; Matin M. A.; Selim M. A.; Al Mutairi M.; Al Nakhli D.; Al Aidaroos A. Y.; et al. Clinical aspects and outcomes of 70 patients with Middle East respiratory syndrome coronavirus infection: a single-center experience in Saudi Arabia. Int. J. Infect. Dis. 2014, 29, 301–306. 10.1016/j.ijid.2014.09.003. 25303830 PMC7110769

[ref93] Al-Hameed F.; Wahla A. S.; Siddiqui S.; Ghabashi A.; Al-Shomrani M.; Al-Thaqafi A.; Tashkandi Y. Characteristics and outcomes of Middle East respiratory syndrome coronavirus patients admitted to an intensive care unit in Jeddah, Saudi Arabia. Journal of intensive care medicine 2016, 31 (5), 344–348. 10.1177/0885066615579858. 25862629

[ref94] Xu P.; Sun G.-D.; Li Z.-Z. Clinical Characteristics of Two Human to Human Transmitted Coronaviruses: Corona Virus Disease 2019 versus Middle East Respiratory Syndrome Coronavirus. medRxiv, 2020, 10.1101/2020.03.08.20032821. 32495918

[ref95] Arabi Y. M.; Al-Omari A.; Mandourah Y.; Al-Hameed F.; Sindi A. A.; Alraddadi B.; Shalhoub S.; Almotairi A.; Al Khatib K.; Abdulmomen A.; et al. Critically ill patients with the Middle East respiratory syndrome: a multicenter retrospective cohort study. Crit. Care Med. 2017, 45 (10), 1683–1695. 10.1097/CCM.0000000000002621. 28787295

[ref96] Arabi Y. M.; Arifi A. A.; Balkhy H. H.; Najm H.; Aldawood A. S.; Ghabashi A.; Hawa H.; Alothman A.; Khaldi A.; Al Raiy B. Clinical course and outcomes of critically ill patients with Middle East respiratory syndrome coronavirus infection. Ann. Intern. Med. 2014, 160 (6), 389–397. 10.7326/M13-2486. 24474051

[ref97] Chen N.; Zhou M.; Dong X.; Qu J.; Gong F.; Han Y.; Qiu Y.; Wang J.; Liu Y.; Wei Y.; et al. Epidemiological and clinical characteristics of 99 cases of 2019 novel coronavirus pneumonia in Wuhan, China: a descriptive study. Lancet 2020, 395 (10223), 507–513. 10.1016/S0140-6736(20)30211-7. 32007143 PMC7135076

[ref98] Ali N. Is SARS-CoV-2 associated with liver dysfunction in COVID-19 patients?. Clin. Res. Hepatol. Gastroenterol. 2020, 44, e84 10.1016/j.clinre.2020.05.002. 32471656 PMC7241387

[ref99] Bruisten S.; Nilsson-Ihrfelt E.; Buhrman M.; Ekselius L. TOXBASE. Emerg Med. J. 2006 Aug; 23 (8): 614–7. PMID: 16858093 [PubMed-in process] 24: Team V, Markovic M. Internet advertising of artificial tanning in Australia. Oncol. Nurs. Forum 2006, 249–254.16518440

[ref100] Yang Z.; Xu M.; Yi J.; Jia W. Clinical characteristics and mechanism of liver damage in patients with severe acute respiratory syndrome. HBPD INT 2005, 4 (1), 60–63. 15730921

[ref101] Yeung M.-L.; Yao Y.; Jia L.; Chan J. F.; Chan K.-H.; Cheung K.-F.; Chen H.; Poon V. K.; Tsang A. K.; To K. K.; et al. MERS coronavirus induces apoptosis in kidney and lung by upregulating Smad7 and FGF2. Nature microbiology 2016, 1 (3), 1–8. 10.1038/nmicrobiol.2016.4. PMC7097571 27572168

[ref102] Lely A.; Hamming I.; van Goor H.; Navis G. Renal ACE2 expression in human kidney disease. J. Pathol. 2004, 204 (5), 587–593. 10.1002/path.1670. 15538735

[ref103] Henry B. M.; Lippi G. Chronic kidney disease is associated with severe coronavirus disease 2019 (COVID-19) infection. Int. Urol. Nephrol. 2020, 52 (6), 1193–1194. 10.1007/s11255-020-02451-9. 32222883 PMC7103107

[ref104] Martinez-Rojas M. A.; Vega-Vega O.; Bobadilla N. A. Is the kidney a target of SARS-CoV-2. American Journal of Physiology-Renal Physiology 2020, 318 (6), F1454–F1462. 10.1152/ajprenal.00160.2020. 32412303 PMC7303722

[ref105] Falsey A. R. Respiratory viral infections. In Genomic and Precision Medicine; Elsevier, 2019; pp 117–139.

[ref106] Cobo F. Suppl 1: application of molecular diagnostic techniques for viral testing. Open Virol. J. 2012, 5, 104 10.2174/1874357901206010104. PMC3522074 23248732

[ref107] Zhang W.; Du R.-H.; Li B.; Zheng X.-S.; Yang X.-L.; Hu B.; Wang Y.-Y.; Xiao G.-F.; Yan B.; Shi Z.-L.; Zhou P. Molecular and serological investigation of 2019-nCoV infected patients: implication of multiple shedding routes. Emerging Microbes Infect. 2020, 9 (1), 386–389. 10.1080/22221751.2020.1729071. PMC7048229 32065057

[ref108] Rappe J. C.; García-Nicolás O.; Flückiger F.; Thür B.; Hofmann M. A.; Summerfield A.; Ruggli N. Heterogeneous antigenic properties of the porcine reproductive and respiratory syndrome virus nucleocapsid. Veterinary research 2016, 47 (1), 117 10.1186/s13567-016-0399-9. 27871316 PMC5118883

[ref109] Shukla S.; Hong S.-Y.; Chung S. H.; Kim M. Rapid detection strategies for the global threat of Zika virus: current state, new hypotheses, and limitations. Front. Microbiol. 2016, 7, 1685 10.3389/fmicb.2016.01685. 27822207 PMC5075579

[ref110] Zherdev A. V.; Dzantiev B. B. Ways to reach lower detection limits of lateral flow immunoassays. Rapid Tests: Advances in Design, Format and Diagnostic Applications; Anfossi L., Ed.; InTechOpen: London, 2018; pp 9–43.

[ref111] Liu Y.; Deng Y.; Dong H.; Liu K.; He N. Progress on sensors based on nanomaterials for rapid detection of heavy metal ions. Sci. China: Chem. 2017, 60 (3), 329–337. 10.1007/s11426-016-0253-2.

[ref112] Torres-Sangiao E.; Holban A. M.; Gestal M. C. Advanced nanobiomaterials: vaccines, diagnosis and treatment of infectious diseases. Molecules 2016, 21 (7), 867 10.3390/molecules21070867. PMC6273484 27376260

[ref113] Sykora S.; Correro M. R.; Moridi N.; Belliot G.; Pothier P.; Dudal Y.; Corvini P. F. X.; Shahgaldian P. A Biocatalytic Nanomaterial for the Label-Free Detection of Virus-Like Particles. ChemBioChem 2017, 18 (11), 996–1000. 10.1002/cbic.201700126. 28297127

[ref114] Hematian A.; Sadeghifard N.; Mohebi R.; Taherikalani M.; Nasrolahi A.; Amraei M.; Ghafourian S. Traditional and modern cell culture in virus diagnosis. Osong public health and research perspectives 2016, 7 (2), 77–82. 10.1016/j.phrp.2015.11.011. 27169004 PMC4850366

[ref115] Sridhar S.; To K. K.; Chan J. F.; Lau S. K.; Woo P. C.; Yuen K.-Y. A systematic approach to novel virus discovery in emerging infectious disease outbreaks. J. Mol. Diagn. 2015, 17 (3), 230–241. 10.1016/j.jmoldx.2014.12.002. 25746799 PMC7106266

[ref116] Soler M. Laboratory diagnosis to dengue virus infections. Acta científica venezolana 1998, 49, 25. 10030051

[ref117] Diel D.; Lawson S.; Okda F.; Singrey A.; Clement T.; Fernandes M.; Christopher-Hennings J.; Nelson E. Porcine epidemic diarrhea virus: an overview of current virological and serological diagnostic methods. Virus Res. 2016, 226, 60–70. 10.1016/j.virusres.2016.05.013. 27189041 PMC7172987

[ref118] Balasuriya U. B.; Crossley B. M.; Timoney P. J. A review of traditional and contemporary assays for direct and indirect detection of Equid herpesvirus 1 in clinical samples. J. Vet. Diagn. Invest. 2015, 27 (6), 673–687. 10.1177/1040638715605558. 26472746

[ref119] Azmi A.; Amsyar Azman A.; Ibrahim S.; Md Yunus M. A. Techniques in Advancing the Capabilities of Various Nitrate Detection Methods: A Review. Int. J. Smart Sens. Intell. Syst. 2017, 10 (2), 223 10.21307/ijssis-2017-210.

[ref120] Amer H.; Abd El Wahed A.; Shalaby M.; Almajhdi F.; Hufert F.; Weidmann M. A new approach for diagnosis of bovine coronavirus using a reverse transcription recombinase polymerase amplification assay. J. Virol. Methods 2013, 193 (2), 337–340. 10.1016/j.jviromet.2013.06.027. 23811231 PMC7113639

[ref121] Nguyen T.; Duong Bang D.; Wolff A. 2019 novel coronavirus disease (COVID-19): paving the road for rapid detection and point-of-care diagnostics. Micromachines 2020, 11 (3), 306 10.3390/mi11030306. PMC7142866 32183357

[ref122] Lee J.; Morita M.; Takemura K.; Park E. Y. A multi-functional gold/iron-oxide nanoparticle-CNT hybrid nanomaterial as virus DNA sensing platform. Biosens. Bioelectron. 2018, 102, 425–431. 10.1016/j.bios.2017.11.052. 29175218

[ref123] Kilianski A.; Roth P. A.; Liem A. T.; Hill J. M.; Willis K. L.; Rossmaier R. D.; Marinich A. V.; Maughan M. N.; Karavis M. A.; Kuhn J. H.; et al. Use of unamplified RNA/cDNA–hybrid nanopore sequencing for rapid detection and characterization of RNA viruses. Emerging Infect. Dis. 2016, 22 (8), 1448 10.3201/eid2208.160270. PMC4982148 27191483

[ref124] Mehrabani S.; Maker A. J.; Armani A. M. Hybrid integrated label-free chemical and biological sensors. Sensors 2014, 14 (4), 5890–5928. 10.3390/s140405890. 24675757 PMC4029679

[ref125] Roh C. A facile inhibitor screening of SARS coronavirus N protein using nanoparticle-based RNA oligonucleotide. Int. J. Nanomed. 2012, 7, 2173 10.2147/IJN.S31379. PMC3356205 22619553

[ref126] Chen Y.; Chan K.-H.; Hong C.; Kang Y.; Ge S.; Chen H.; Wong E. Y.; Joseph S.; Patteril N. G.; Wernery U.; et al. A highly specific rapid antigen detection assay for on-site diagnosis of MERS. J. Infect. 2016, 73 (1), 82 10.1016/j.jinf.2016.04.014. 27144915 PMC7127149

[ref127] Teengam P.; Siangproh W.; Tuantranont A.; Vilaivan T.; Chailapakul O.; Henry C. S. Multiplex paper-based colorimetric DNA sensor using pyrrolidinyl peptide nucleic acid-induced AgNPs aggregation for detecting MERS-CoV, MTB, and HPV oligonucleotides. Anal. Chem. 2017, 89 (10), 5428–5435. 10.1021/acs.analchem.7b00255. 28394582 PMC7077925

[ref128] Ahmed S. R.; Nagy É.; Neethirajan S. Self-assembled star-shaped chiroplasmonic gold nanoparticles for an ultrasensitive chiro-immunosensor for viruses. RSC Adv. 2017, 7 (65), 40849–40857. 10.1039/C7RA07175B.

[ref129] Wang K.; Zhu J.; Dong H.; Pei Z.; Zhou T.; Hu G. Rapid Detection of Variant and Classical Porcine Epidemic Diarrhea Virus by Nano-Nest PCR. Pakistan Veterinary Journal 2017, 37 (2), 225.

[ref130] Ahmed S. R.; Kang S. W.; Oh S.; Lee J.; Neethirajan S. Chiral zirconium quantum dots: a new class of nanocrystals for optical detection of coronavirus. Heliyon 2018, 4 (8), e00766 10.1016/j.heliyon.2018.e00766. 30186985 PMC6120744

[ref131] Weng X.; Neethirajan S. Immunosensor based on antibody-functionalized MoS 2 for rapid detection of avian coronavirus on cotton thread. IEEE Sens. J. 2018, 18 (11), 4358–4363. 10.1109/JSEN.2018.2829084. 32390783 PMC7186039

[ref132] Kim H.; Park M.; Hwang J.; Kim J. H.; Chung D.-R.; Lee K.-s.; Kang M. Development of label-free colorimetric assay for MERS-CoV using gold nanoparticles. ACS sensors 2019, 4 (5), 1306–1312. 10.1021/acssensors.9b00175. 31062580

[ref133] Liu I.-L.; Lin Y.-C.; Lin Y.-C.; Jian C.-Z.; Cheng I.-C.; Chen H.-W. A novel immunochromatographic strip for antigen detection of avian infectious bronchitis virus. Int. J. Mol. Sci. 2019, 20 (9), 2216 10.3390/ijms20092216. PMC6540333 31064083

[ref134] Udugama B.; Kadhiresan P.; Kozlowski H. N.; Malekjahani A.; Osborne M.; Li V. Y.; Chen H.; Mubareka S.; Gubbay J. B.; Chan W. C. Diagnosing COVID-19: the disease and tools for detection. ACS Nano 2020, 14 (4), 3822–3835. 10.1021/acsnano.0c02624. 32223179

[ref135] Xiang J.; Yan M.; Li H.; Liu T.; Lin C.; Huang S.; Shen C. Evaluation of Enzyme-Linked Immunoassay and Colloidal Gold-Immunochromatographic Assay Kit for Detection of Novel Coronavirus (SARS-Cov-2) Causing an Outbreak of Pneumonia (COVID-19). MedRxiv, 2020, 10.1101/2020.02.27.20028787.

[ref136] Gorshkov K.; Susumu K.; Chen J.; Xu M.; Pradhan M.; Zhu W.; Hu X.; Breger J. C.; Wolak M.; Oh E. Quantum dot-conjugated sars-cov-2 spike pseudo-virions enable tracking of angiotensin converting enzyme 2 binding and endocytosis. ACS Nano 2020, 14 (9), 12234–12247. 10.1021/acsnano.0c05975. 32845122 PMC7482579

[ref137] Seo G.; Lee G.; Kim M. J.; Baek S.-H.; Choi M.; Ku K. B.; Lee C.-S.; Jun S.; Park D.; Kim H. G.; et al. Rapid detection of COVID-19 causative virus (SARS-CoV-2) in human nasopharyngeal swab specimens using field-effect transistor-based biosensor. ACS Nano 2020, 14 (4), 5135–5142. 10.1021/acsnano.0c02823. 32293168

[ref138] Nikaeen G.; Abbaszadeh S.; Yousefinejad S. Application of nanomaterials in treatment, anti-infection and detection of coronaviruses. Nanomedicine 2020, 15 (15), 1501–1512. 10.2217/nnm-2020-0117. 32378459 PMC7373208

[ref139] Foudeh A. M.; Fatanat Didar T.; Veres T.; Tabrizian M. Microfluidic designs and techniques using lab-on-a-chip devices for pathogen detection for point-of-care diagnostics. Lab Chip 2012, 12 (18), 3249–3266. 10.1039/c2lc40630f. 22859057

[ref140] Xia Y.; Chen Y.; Tang Y.; Cheng G.; Yu X.; He H.; Cao G.; Lu H.; Liu Z.; Zheng S.-Y. Smartphone-based point-of-care microfluidic platform fabricated with a ZnO nanorod template for colorimetric virus detection. ACS sensors 2019, 4 (12), 3298–3307. 10.1021/acssensors.9b01927. 31769284

[ref141] Shao D.; Li J.; Pan Y.; Zhang X.; Zheng X.; Wang Z.; Zhang M.; Zhang H.; Chen L. Noninvasive theranostic imaging of HSV-TK/GCV suicide gene therapy in liver cancer by folate-targeted quantum dot-based liposomes. Biomater. Sci. 2015, 3 (6), 833–841. 10.1039/C5BM00077G. 26221843

[ref142] Neng J.; Harpster M. H.; Wilson W. C.; Johnson P. A. Surface-enhanced Raman scattering (SERS) detection of multiple viral antigens using magnetic capture of SERS-active nanoparticles. Biosens. Bioelectron. 2013, 41, 316–321. 10.1016/j.bios.2012.08.048. 23021841

[ref143] Pang Y.; Rong Z.; Wang J.; Xiao R.; Wang S. A fluorescent aptasensor for H5N1 influenza virus detection based-on the core–shell nanoparticles metal-enhanced fluorescence (MEF). Biosens. Bioelectron. 2015, 66, 527–532. 10.1016/j.bios.2014.10.052. 25506900

[ref144] Sabela M.; Balme S.; Bechelany M.; Janot J. M.; Bisetty K. A review of gold and silver nanoparticle-based colorimetric sensing assays. Adv. Eng. Mater. 2017, 19 (12), 1700270 10.1002/adem.201700270.

[ref145] Lee C.; Wang P.; Gaston M. A.; Weiss A. A.; Zhang P. Plasmonics-based detection of virus using sialic acid functionalized gold nanoparticles. In Biosensors and Biodetection; Springer, 2017; pp 109–116. 10.1007/978-1-4939-6848-0_7 PMC7121675 28281252

[ref146] Mashhadizadeh M. H.; Pourtaghavi Talemi R. A highly sensitive and selective hepatitis B DNA biosensor using gold nanoparticle electrodeposition on an Au electrode and mercaptobenzaldehyde. Anal. Methods 2014, 6 (22), 8956–8964. 10.1039/C4AY01465K.

[ref147] de la Escosura-Muñiz A.; Maltez-da Costa M.; Sánchez-Espinel C.; Díaz-Freitas B.; Fernández-Suarez J.; González-Fernández Á.; Merkoçi A. Gold nanoparticle-based electrochemical magnetoimmunosensor for rapid detection of anti-hepatitis B virus antibodies in human serum. Biosens. Bioelectron. 2010, 26 (4), 1710–1714. 10.1016/j.bios.2010.07.069. 20724135

[ref148] Ma C.; Xie G.; Zhang W.; Liang M.; Liu B.; Xiang H. Label-free sandwich type of immunosensor for hepatitis C virus core antigen based on the use of gold nanoparticles on a nanostructured metal oxide surface. Microchim. Acta 2012, 178 (3–4), 331–340. 10.1007/s00604-012-0842-1.

[ref149] Hyeon T.; Piao Y.; Park Y. I. Method of preparing iron oxide nanoparticles coated with hydrophilic material, and magnetic resonance imaging contrast agent using the same. Patent US 9352058 B2, 2016.

[ref150] Kamikawa T. L.; Mikolajczyk M. G.; Kennedy M.; Zhang P.; Wang W.; Scott D. E.; Alocilja E. C. Nanoparticle-based biosensor for the detection of emerging pandemic influenza strains. Biosens. Bioelectron. 2010, 26 (4), 1346–1352. 10.1016/j.bios.2010.07.047. 20729069

[ref151] Low S. S.; Tan M. T.; Loh H.-S.; Khiew P. S.; Chiu W. S. Facile hydrothermal growth graphene/ZnO nanocomposite for development of enhanced biosensor. Anal. Chim. Acta 2016, 903, 131–141. 10.1016/j.aca.2015.11.006. 26709306

[ref152] Mao X.; Liu S.; Yang C.; Liu F.; Wang K.; Chen G. Colorimetric detection of hepatitis B virus (HBV) DNA based on DNA-templated copper nanoclusters. Anal. Chim. Acta 2016, 909, 101–108. 10.1016/j.aca.2016.01.009. 26851090

[ref153] Chen X.; Xie H.; Seow Z. Y.; Gao Z. An ultrasensitive DNA biosensor based on enzyme-catalyzed deposition of cupric hexacyanoferrate nanoparticles. Biosens. Bioelectron. 2010, 25 (6), 1420–1426. 10.1016/j.bios.2009.10.041. 19939664

[ref154] Tsang M.-K.; Ye W.; Wang G.; Li J.; Yang M.; Hao J. Ultrasensitive detection of Ebola virus oligonucleotide based on upconversion nanoprobe/nanoporous membrane system. ACS Nano 2016, 10 (1), 598–605. 10.1021/acsnano.5b05622. 26720408

[ref155] Chen C.-C.; Lai Z.-L.; Wang G.-J.; Wu C.-Y. Polymerase chain reaction-free detection of hepatitis B virus DNA using a nanostructured impedance biosensor. Biosens. Bioelectron. 2016, 77, 603–608. 10.1016/j.bios.2015.10.028. 26479905

[ref156] Wen L.; Lin Y.; Zheng Z.-H.; Zhang Z.-L.; Zhang L.-J.; Wang L.-Y.; Wang H.-Z.; Pang D.-W. Labeling the nucleocapsid of enveloped baculovirus with quantum dots for single-virus tracking. Biomaterials 2014, 35 (7), 2295–2301. 10.1016/j.biomaterials.2013.11.069. 24360719

[ref157] Li Y.; Jing L.; Ding K.; Gao J.; Peng Z.; Li Y.; Shen L.; Gao M. Detection of Epstein–Barr virus infection in cancer by using highly specific nanoprobe based on dBSA capped CdTe quantum dots. RSC Adv. 2014, 4 (43), 22545–22550. 10.1039/c4ra02277g.

[ref158] Łoczechin A.; Séron K.; Barras A.; Giovanelli E.; Belouzard S.; Chen Y.-T.; Metzler-Nolte N.; Boukherroub R.; Dubuisson J.; Szunerits S. Functional Carbon Quantum Dots as medical countermeasures to human Coronavirus. ACS Appl. Mater. Interfaces 2019, 11 (46), 42964–42974. 10.1021/acsami.9b15032. 31633330 PMC7075527

[ref159] Chen L.; Qi Z.; Chen R.; Li Y.; Liu S. Sensitive detection of Epstein–Barr virus-derived latent membrane protein 1 based on CdTe quantum dots-capped silica nanoparticle labels. Clin. Chim. Acta 2010, 411 (23–24), 1969–1975. 10.1016/j.cca.2010.08.012. 20713034

[ref160] Riccò R.; Meneghello A.; Enrichi F. Signal enhancement in DNA microarray using dye doped silica nanoparticles: application to human papilloma virus (HPV) detection. Biosens. Bioelectron. 2011, 26 (5), 2761–2765. 10.1016/j.bios.2010.10.024. 21074399

[ref161] Liu F.; Xiang G.; Zhang L.; Jiang D.; Liu L.; Li Y.; Liu C.; Pu X. A novel label free long non-coding RNA electrochemical biosensor based on green L-cysteine electrodeposition and Au–Rh hollow nanospheres as tags. RSC Adv. 2015, 5 (64), 51990–51999. 10.1039/C5RA07904G.

[ref162] Shi L.; Yu Y.; Chen Z.; Zhang L.; He S.; Shi Q.; Yang H. A label-free hemin/G-quadruplex DNAzyme biosensor developed on electrochemically modified electrodes for detection of a HBV DNA segment. RSC Adv. 2015, 5 (15), 11541–11548. 10.1039/C4RA09936B.

[ref163] Liu X.; Cheng Z.; Fan H.; Ai S.; Han R. Electrochemical detection of avian influenza virus H5N1 gene sequence using a DNA aptamer immobilized onto a hybrid nanomaterial-modified electrode. Electrochim. Acta 2011, 56 (18), 6266–6270. 10.1016/j.electacta.2011.05.055.

[ref164] Muti M.; Sharma S.; Erdem A.; Papakonstantinou P. Electrochemical monitoring of nucleic acid hybridization by single-use graphene oxide-based sensor. Electroanalysis 2011, 23 (1), 272–279. 10.1002/elan.201000425.

[ref165] Kim M.-G.; Shon Y.; Lee J.; Byun Y.; Choi B.-S.; Kim Y. B.; Oh Y.-K. Double stranded aptamer-anchored reduced graphene oxide as target-specific nano detector. Biomaterials 2014, 35 (9), 2999–3004. 10.1016/j.biomaterials.2013.12.058. 24418663

[ref166] Bi S.; Zhao T.; Luo B. A graphene oxide platform for the assay of biomolecules based on chemiluminescence resonance energy transfer. Chem. Commun. 2012, 48 (1), 106–108. 10.1039/C1CC15443E. 22037540

[ref167] Liu F.; Kim Y. H.; Cheon D. S.; Seo T. S. Micropatterned reduced graphene oxide based field-effect transistor for real-time virus detection. Sens. Actuators, B 2013, 186, 252–257. 10.1016/j.snb.2013.05.097.

[ref168] Wu Y.-M.; Cen Y.; Huang L.-J.; Yu R.-Q.; Chu X. Upconversion fluorescence resonance energy transfer biosensor for sensitive detection of human immunodeficiency virus antibodies in human serum. Chem. Commun. 2014, 50 (36), 4759–4762. 10.1039/C4CC00569D. 24686326

[ref169] Calderón L.; Harris R.; Cordoba-Diaz M.; Elorza M.; Elorza B.; Lenoir J.; Adriaens E.; Remon J.; Heras A.; Cordoba-Diaz D. Nano and microparticulate chitosan-based systems for antiviral topical delivery. Eur. J. Pharm. Sci. 2013, 48 (1–2), 216–222. 10.1016/j.ejps.2012.11.002. 23159663

[ref170] Cho I. H.; Lee D. G.; Yang Y. Y. Composition with sterilizing activity against bacteria, fungus and viruses, application thereof and method for preparation thereof. Patents US 2013012980 A1, 2014.

[ref171] Lv X.; Wang P.; Bai R.; Cong Y.; Suo S.; Ren X.; Chen C. Inhibitory effect of silver nanomaterials on transmissible virus-induced host cell infections. Biomaterials 2014, 35 (13), 4195–4203. 10.1016/j.biomaterials.2014.01.054. 24524838 PMC7112386

[ref172] Chen Y.-N.; Hsueh Y.-H.; Hsieh C.-T.; Tzou D.-Y.; Chang P.-L. Antiviral activity of graphene–silver nanocomposites against non-enveloped and enveloped viruses. Int. J. Environ. Res. Public Health 2016, 13 (4), 430 10.3390/ijerph13040430. 27104546 PMC4847092

[ref173] Hu C.-M. J.; Chang W.-S.; Fang Z.-S.; Chen Y.-T.; Wang W.-L.; Tsai H.-H.; Chueh L.-L.; Takano T.; Hohdatsu T.; Chen H.-W. Nanoparticulate vacuolar ATPase blocker exhibits potent host-targeted antiviral activity against feline coronavirus. Sci. Rep. 2017, 7 (1), 1–11. 10.1038/s41598-017-13316-0. 29026122 PMC5638965

[ref174] Ciejka J.; Wolski K.; Nowakowska M.; Pyrc K.; Szczubiałka K. Biopolymeric nano/microspheres for selective and reversible adsorption of coronaviruses. Mater. Sci. Eng., C 2017, 76, 735–742. 10.1016/j.msec.2017.03.047. PMC7126271 28482585

[ref175] Du T.; Liang J.; Dong N.; Lu J.; Fu Y.; Fang L.; Xiao S.; Han H. Glutathione-capped Ag2S nanoclusters inhibit coronavirus proliferation through blockage of viral RNA synthesis and budding. ACS Appl. Mater. Interfaces 2018, 10 (5), 4369–4378. 10.1021/acsami.7b13811. 29337529

[ref176] Zheng Z.; Li Z.; Xu C.; Guo B.; Guo P. Folate-displaying exosome mediated cytosolic delivery of siRNA avoiding endosome trapping. J. Controlled Release 2019, 311, 43–49. 10.1016/j.jconrel.2019.08.021. PMC6874920 31446085

[ref177] Arbuthnot P. Gene Therapy for Respiratory Viral Infections. Gene Therapy for Viral Infections 2015, 281 10.1016/B978-0-12-410518-8.00009-0.

[ref178] Rabaan A. A.; Al-Ahmed S. H.; Haque S.; Sah R.; Tiwari R.; Malik Y. S.; Dhama K.; Yatoo M. I.; Bonilla-Aldana D. K.; Rodriguez-Morales A. J. SARS-CoV-2, SARS-CoV, and MERS-COV: a comparative overview. Infez Med. 2020, 28 (2), 174–184. 32275259

[ref179] Gu S. H.; Yu C. H.; Song Y.; Kim N. Y.; Sim E.; Choi J. Y.; Song D. H.; Hur G. H.; Shin Y. K.; Jeong S. T. A Small interfering RNA lead targeting RNA-dependent RNA-polymerase effectively inhibit the SARS-CoV-2 infection in Golden Syrian hamster and Rhesus macaque. bioRxiv, 2020, 10.1101/2020.07.07.190967.

[ref180] Wu C.-J.; Huang H.-W.; Liu C.-Y.; Hong C.-F.; Chan Y.-L. Inhibition of SARS-CoV replication by siRNA. Antiviral Res. 2005, 65 (1), 45–48. 10.1016/j.antiviral.2004.09.005. 15652970 PMC7114151

[ref181] Zhang Y.; Li T.; Fu L.; Yu C.; Li Y.; Xu X.; Wang Y.; Ning H.; Zhang S.; Chen W.; et al. Silencing SARS-CoV Spike protein expression in cultured cells by RNA interference. FEBS Lett. 2004, 560 (1-3), 141–146. 10.1016/S0014-5793(04)00087-0. 14988013 PMC7127813

[ref182] Tang Q.; Li B.; Woodle M.; Lu P. Y. Application of siRNA against SARS in the rhesus macaque model. In RNAi; Springer, 2008; pp 139–158. 10.1007/978-1-59745-191-8_11 PMC7121115 18369784

[ref183] Li B.-j.; Tang Q.; Cheng D.; Qin C.; Xie F. Y.; Wei Q.; Xu J.; Liu Y.; Zheng B.-j.; Woodle M. C.; et al. Using siRNA in prophylactic and therapeutic regimens against SARS coronavirus in Rhesus macaque. Nat. Med. 2005, 11 (9), 944–951. 10.1038/nm1280. 16116432 PMC7095788

[ref184] Donia A.; Bokhari H. RNA interference as a promising treatment against SARS-CoV-2. Int. Microbiol. 2021, 24, 123–124. 10.1007/s10123-020-00146-w. 32875426 PMC7462657

[ref185] Tatiparti K.; Sau S.; Kashaw S. K.; Iyer A. K. siRNA delivery strategies: a comprehensive review of recent developments. Nanomaterials 2017, 7 (4), 77 10.3390/nano7040077. PMC5408169 28379201

[ref186] Gavrilov K.; Saltzman W. M. Therapeutic siRNA: principles, challenges, and strategies. Yale J. Biol. Med. 2012, 85 (2), 187. 22737048 PMC3375670

[ref187] Mahmoodi Chalbatani G.; Dana H.; Gharagouzloo E.; Grijalvo S.; Eritja R.; Logsdon C. D.; Memari F.; Miri S. R.; Rezvani Rad M.; Marmari V. Small interfering RNAs (siRNAs) in cancer therapy: a nano-based approach. Int. J. Nanomed. 2019, 14, 3111 10.2147/IJN.S200253. PMC6504672 31118626

[ref188] Ho W.; Zhang X. Q.; Xu X. Biomaterials in siRNA delivery: a comprehensive review. Adv. Healthcare Mater. 2016, 5 (21), 2715–2731. 10.1002/adhm.201600418. 27700013

[ref189] Khademhosseini A.; Peppas N. A. Adv. Healthcare Mater. 2013, 2 (1), 10–12. 10.1002/adhm.201200444. 23299936

[ref190] Artiga A.; Serrano-Sevilla I.; De Matteis L.; Mitchell S. G.; de la Fuente J. M. Current status and future perspectives of gold nanoparticle vectors for siRNA delivery. J. Mater. Chem. B 2019, 7 (6), 876–896. 10.1039/C8TB02484G. 32255093

[ref191] Sohail M. F.; Hussain S. Z.; Saeed H.; Javed I.; Sarwar H. S.; Nadhman A.; Rehman M.; Jahan S.; Hussain I.; Shahnaz G. Polymeric nanocapsules embedded with ultra-small silver nanoclusters for synergistic pharmacology and improved oral delivery of Docetaxel. Sci. Rep. 2018, 8 (1), 1–11. 10.1038/s41598-018-30749-3. 30190588 PMC6127092

[ref192] Chen C.-K.; Huang P.-K.; Law W.-C.; Chu C.-H.; Chen N.-T.; Lo L.-W. Biodegradable polymers for gene-delivery applications. Int. J. Nanomed. 2020, 15, 2131 10.2147/IJN.S222419. PMC7125329 32280211

[ref193] Kamaly N.; Yameen B.; Wu J.; Farokhzad O. C. Degradable controlled-release polymers and polymeric nanoparticles: mechanisms of controlling drug release. Chem. Rev. 2016, 116 (4), 2602–2663. 10.1021/acs.chemrev.5b00346. 26854975 PMC5509216

[ref194] Shajari N.; Mansoori B.; Davudian S.; Mohammadi A.; Baradaran B. Overcoming the challenges of siRNA delivery: nanoparticle strategies. Curr. Drug Delivery 2017, 14 (1), 36–46. 10.2174/1567201813666160816105408. 27538460

[ref195] Dabbagh A.; Abu Kasim N. H.; Yeong C. H.; Wong T. W.; Abdul Rahman N. Critical parameters for particle-based pulmonary delivery of chemotherapeutics. J. Aerosol Med. Pulm. Drug Delivery 2018, 31 (3), 139–154. 10.1089/jamp.2017.1382. 29022837

[ref196] Thanki K.; van Eetvelde D.; Geyer A.; Fraire J.; Hendrix R.; Van Eygen H.; Putteman E.; Sami H.; de Souza Carvalho-Wodarz C.; Franzyk H.; et al. Mechanistic profiling of the release kinetics of siRNA from lipidoid-polymer hybrid nanoparticles in vitro and in vivo after pulmonary administration. J. Controlled Release 2019, 310, 82–93. 10.1016/j.jconrel.2019.08.004. 31398360

[ref197] Thanh Le T.; Andreadakis Z.; Kumar A.; Gomez Roman R.; Tollefsen S.; Saville M.; Mayhew S. The COVID-19 vaccine development landscape. Nat. Rev. Drug Discovery 2020, 19 (5), 305–306. 10.1038/d41573-020-00073-5. 32273591

[ref198] Eygeris Y.; Patel S.; Jozic A.; Sahay G. Deconvoluting Lipid Nanoparticle Structure for Messenger RNA Delivery. Nano Lett. 2020, 20 (6), 4543–4549. 10.1021/acs.nanolett.0c01386. 32375002

[ref199] Uludağ H.; Parent K.; Aliabadi H. M.; Haddadi A. Prospects for RNAi Therapy of COVID-19. Front. Bioeng. Biotechnol. 2020, 8, 916 10.3389/fbioe.2020.00916. 32850752 PMC7409875

[ref200] Ullah A.; Qazi J.; Rahman L.; Kanaras A. G.; Khan W. S.; Hussain I.; Rehman A. Nanoparticles-assisted delivery of antiviral-siRNA as inhalable treatment for human respiratory viruses: A candidate approach against SARS-COV-2. Nano Select 2020, 1 (6), 612–621. 10.1002/nano.202000125. PMC7675679 34485978

[ref201] Totura A. L.; Baric R. S. SARS coronavirus pathogenesis: host innate immune responses and viral antagonism of interferon. Curr. Opin. Virol. 2012, 2 (3), 264–275. 10.1016/j.coviro.2012.04.004. 22572391 PMC7102726

[ref202] Cervantes-Barragan L.; Züst R.; Weber F.; Spiegel M.; Lang K. S.; Akira S.; Thiel V.; Ludewig B. Control of coronavirus infection through plasmacytoid dendritic-cell–derived type I interferon. Blood 2007, 109 (3), 1131–1137. 10.1182/blood-2006-05-023770. 16985170 PMC8254533

[ref203] Davidson S.; Maini M. K.; Wack A. Disease-promoting effects of type I interferons in viral, bacterial, and coinfections. J. Interferon Cytokine Res. 2015, 35 (4), 252–264. 10.1089/jir.2014.0227. 25714109 PMC4389918

[ref204] Newton A. H.; Cardani A.; Braciale T. J. The host immune response in respiratory virus infection: balancing virus clearance and immunopathology. Semin. Immunopathol. 2016, 38, 471–482. 10.1007/s00281-016-0558-0. 26965109 PMC4896975

[ref205] Peng Y.; Mentzer A. J.; Liu G.; Yao X.; Yin Z.; Dong D.; Dejnirattisai W.; Rostron T.; Supasa P.; Liu C. Broad and strong memory CD4+ and CD8+ T cells induced by SARS-CoV-2 in UK convalescent individuals following COVID-19. Nature immunology 2020, 21, 1336–1345. 10.1038/s41590-020-0782-6. 32887977 PMC7611020

[ref206] SARS-CoV-2: a storm is raging. J. Clin. Invest. 2020, 130 (5), 2202 10.1021/acsbiomaterials.1c00318. 32217834 PMC7190904

[ref207] Giménez E.; Albert E.; Torres I.; Remigia M. J.; Alcaraz M. J.; Galindo M. J.; Blasco M. L.; Solano C.; Forner M. J.; Redón J.; et al. SARS-CoV-2-reactive interferon-γ-producing CD8+ T cells in patients hospitalized with coronavirus disease 2019. J. Med. Virol. 2021, 93 (1), 375–382. 10.1002/jmv.26213. 32579268 PMC7361624

[ref208] Lei X.; Dong X.; Ma R.; Wang W.; Xiao X.; Tian Z.; Wang C.; Wang Y.; Li L.; Ren L.; et al. Activation and evasion of type I interferon responses by SARS-CoV-2. Nat. Commun. 2020, 11 (1), 1–12. 10.1038/s41467-020-17665-9. 32733001 PMC7392898

[ref209] Rabouw H. H.; Langereis M. A.; Knaap R. C. M.; Dalebout T. J.; Canton J.; Sola I.; Enjuanes L.; Bredenbeek P. J.; Kikkert M.; de Groot R. J.; van Kuppeveld F. J. M. Middle East respiratory coronavirus accessory protein 4a inhibits PKR-mediated antiviral stress responses. PLoS Pathog. 2016, 12 (10), e1005982 10.1371/journal.ppat.1005982. 27783669 PMC5081173

[ref210] Gutierrez-Alvarez J.; Wang L.; Fernandez-Delgado R.; Li K.; McCray P. B.; Perlman S.; Sola I.; Zuñiga S.; Enjuanes L. Middle East respiratory syndrome coronavirus gene 5 modulates pathogenesis in mice. J. Virol. 2021, 95 (3), e01172-20 10.1128/JVI.01172-20. 33144319 PMC7925085

[ref211] Prompetchara E.; Ketloy C.; Palaga T. Immune responses in COVID-19 and potential vaccines: Lessons learned from SARS and MERS epidemic. Asian Pac J. Allergy Immunol 2020, 38 (1), 1–9. 10.12932/AP-200220-0772. 32105090

[ref212] Teijaro J. R. Type I interferons in viral control and immune regulation. Curr. Opin. Virol. 2016, 16, 31–40. 10.1016/j.coviro.2016.01.001. 26812607 PMC4821698

[ref213] Kuri T.; Zhang X.; Habjan M.; Martínez-Sobrido L.; García-Sastre A.; Yuan Z.; Weber F. Interferon priming enables cells to partially overturn the SARS coronavirus-induced block in innate immune activation. J. Gen. Virol. 2009, 90 (11), 2686 10.1099/vir.0.013599-0. 19625461 PMC2888313

[ref214] Lokugamage K. G.; Hage A.; Schindewolf C.; Rajsbaum R.; Menachery V. D. SARS-CoV-2 is sensitive to type I interferon pretreatment. BioRxiv, 2020, 10.1101/2020.03.07.982264. PMC7654262 32938761

[ref215] Keam S.; Megawati D.; Patel S. K.; Tiwari R.; Dhama K.; Harapan H. Immunopathology and immunotherapeutic strategies in severe acute respiratory syndrome coronavirus 2 infection. Rev. Med. Virol. 2020, 30 (5), e2123 10.1002/rmv.2123. 32648313 PMC7404843

[ref216] Kikkert M. Innate immune evasion by human respiratory RNA viruses. J. Innate Immun. 2020, 12 (1), 4–20. 10.1159/000503030. 31610541 PMC6959104

[ref217] Li X.; Geng M.; Peng Y.; Meng L.; Lu S. Molecular immune pathogenesis and diagnosis of COVID-19. J. Pharm. Anal. 2020, 10 (2), 102–108. 10.1016/j.jpha.2020.03.001. 32282863 PMC7104082

[ref218] Dhama K.; Sharun K.; Tiwari R.; Dadar M.; Malik Y. S.; Singh K. P.; Chaicumpa W. COVID-19, an emerging coronavirus infection: advances and prospects in designing and developing vaccines, immunotherapeutics, and therapeutics. Hum. Vaccines Immunother. 2020, 16 (6), 1232–1238. 10.1080/21645515.2020.1735227. PMC7103671 32186952

[ref219] AminJafari A.; Ghasemi S. The possible of immunotherapy for COVID-19: A systematic review. Int. Immunopharmacol. 2020, 83, 106455 10.1016/j.intimp.2020.106455. 32272396 PMC7128194

[ref220] Casadevall A.; Pirofski L. The convalescent sera option for containing COVID-19. J. Clin. Invest. 2020, 130, 1545 10.1172/JCI138003. 32167489 PMC7108922

[ref221] Liu L.; Wang P.; Nair M. S.; Yu J.; Rapp M.; Wang Q.; Luo Y.; Chan J. F.-W.; Sahi V.; Figueroa A.; et al. Potent neutralizing antibodies against multiple epitopes on SARS-CoV-2 spike. Nature 2020, 584 (7821), 450–456. 10.1038/s41586-020-2571-7. 32698192

[ref222] Wec A. Z.; Wrapp D.; Herbert A. S.; Maurer D. P.; Haslwanter D.; Sakharkar M.; Jangra R. K.; Dieterle M. E.; Lilov A.; Huang D.; et al. Broad neutralization of SARS-related viruses by human monoclonal antibodies. Science 2020, 369 (6504), 731–736. 10.1126/science.abc7424. 32540900 PMC7299279

[ref223] Weisblum Y.; Schmidt F.; Zhang F.; DaSilva J.; Poston D.; Lorenzi J. C.; Muecksch F.; Rutkowska M.; Hoffmann H.-H.; Michailidis E.; et al. Escape from neutralizing antibodies by SARS-CoV-2 spike protein variants. eLife 2020, 9, e61312 10.7554/eLife.61312. 33112236 PMC7723407

[ref224] Felgenhauer U.; Schoen A.; Gad H. H.; Hartmann R.; Schaubmar A. R.; Failing K.; Drosten C.; Weber F. Inhibition of SARS–CoV-2 by type I and type III interferons. J. Biol. Chem. 2020, 295 (41), 13958–13964. 10.1074/jbc.AC120.013788. 32587093 PMC7549028

[ref225] Falzarano D.; De Wit E.; Martellaro C.; Callison J.; Munster V. J.; Feldmann H. Inhibition of novel β coronavirus replication by a combination of interferon-α2b and ribavirin. Sci. Rep. 2013, 3, 1686 10.1038/srep01686. 23594967 PMC3629412

[ref226] Zielecki F.; Weber M.; Eickmann M.; Spiegelberg L.; Zaki A. M.; Matrosovich M.; Becker S.; Weber F. Human cell tropism and innate immune system interactions of human respiratory coronavirus EMC compared to those of severe acute respiratory syndrome coronavirus. Journal of virology 2013, 87 (9), 5300–5304. 10.1128/JVI.03496-12. 23449793 PMC3624328

[ref227] Anders K. L.; Indriani C.; Ahmad R. A.; Tantowijoyo W.; Arguni E.; Andari B.; Jewell N. P.; Rances E.; O’Neill S. L.; Simmons C. P.; Utarini A. The AWED trial (Applying Wolbachia to Eliminate Dengue) to assess the efficacy of Wolbachia-infected mosquito deployments to reduce dengue incidence in Yogyakarta, Indonesia: study protocol for a cluster randomised controlled trial. Trials 2018, 19 (1), 1–16. 10.1186/s13063-018-2670-z. 29855331 PMC5984439

[ref228] Wang Z.; Yang B.; Li Q.; Wen L.; Zhang R. Clinical features of 69 cases with coronavirus disease 2019 in Wuhan, China. Clin. Infect. Dis. 2020, 71 (15), 769–777. 10.1093/cid/ciaa272. 32176772 PMC7184452

[ref229] Jodele S.; Köhl J. Tackling COVID-19 infection through complement-targeted immunotherapy. Br. J. Pharmacol. 2020, 10.1111/bph.15187. PMC7361469 32643798

[ref230] Zhang Q.; Wang Y.; Qi C.; Shen L.; Li J. Clinical trial analysis of 2019-nCoV therapy registered in China. J. Med. Virol. 2020, 92 (6), 540–545. 10.1002/jmv.25733. 32108352 PMC7228274

[ref231] Levine M. M. Monoclonal antibody therapy for Ebola virus disease. N. Engl. J. Med. 2019, 381, 2365–2366. 10.1056/NEJMe1915350. 31774948

[ref232] Di Gioacchino M.; Petrarca C.; Gatta A.; Scarano G.; Farinelli A.; Della Valle L.; Lumaca A.; Del Biondo P.; Paganelli R.; Di Giampaolo L. Nanoparticle-based immunotherapy: state of the art and future perspectives. Expert Rev. Clin. Immunol. 2020, 16 (5), 513–525. 10.1080/1744666X.2020.1762572. 32343153

[ref233] Shi Y.; Lammers T. Combining nanomedicine and immunotherapy. Acc. Chem. Res. 2019, 52 (6), 1543–1554. 10.1021/acs.accounts.9b00148. 31120725 PMC7115879

[ref234] Irvine D. J.; Hanson M. C.; Rakhra K.; Tokatlian T. Synthetic nanoparticles for vaccines and immunotherapy. Chem. Rev. 2015, 115 (19), 11109–11146. 10.1021/acs.chemrev.5b00109. 26154342 PMC4688911

[ref235] Wilson J. T.; Keller S.; Manganiello M. J.; Cheng C.; Lee C.-C.; Opara C.; Convertine A.; Stayton P. S. pH-Responsive nanoparticle vaccines for dual-delivery of antigens and immunostimulatory oligonucleotides. ACS Nano 2013, 7 (5), 3912–3925. 10.1021/nn305466z. 23590591 PMC4042837

[ref236] Sayour E. J.; De Leon G.; Pham C.; Grippin A.; Kemeny H.; Chua J.; Huang J.; Sampson J. H.; Sanchez-Perez L.; Flores C.; Mitchell D. A. Systemic activation of antigen-presenting cells via RNA-loaded nanoparticles. Oncoimmunology 2017, 6 (1), e1256527 10.1080/2162402X.2016.1256527. 28197373 PMC5283636

[ref237] Dykman L.; Khlebtsov N. Gold nanoparticles in biology and medicine: recent advances and prospects. Acta Nat. 2011, 3 (2), 34 10.32607/20758251-2011-3-2-34-55. PMC3347577 22649683

[ref238] Yang Z.; Ma Y.; Zhao H.; Yuan Y.; Kim B. Y. Nanotechnology platforms for cancer immunotherapy. Wiley Interdiscip. Rev.: Nanomed. Nanobiotechnol. 2020, 12 (2), e1590 10.1002/wnan.1590. 31696664

[ref239] Afrough B.; Dowall S.; Hewson R. Emerging viruses and current strategies for vaccine intervention. Clin. Exp. Immunol. 2019, 196 (2), 157–166. 10.1111/cei.13295. 30993690 PMC6468171

[ref240] Zaman M.; Good M. F.; Toth I. Nanovaccines and their mode of action. Methods 2013, 60 (3), 226–231. 10.1016/j.ymeth.2013.04.014. 23623821

[ref241] Gregory A. E.; Williamson D.; Titball R. Vaccine delivery using nanoparticles. Front. Cell. Infect. Microbiol. 2013, 3, 13 10.3389/fcimb.2013.00013. 23532930 PMC3607064

[ref242] Staroverov S.; Vidyasheva I.; Gabalov K.; Vasilenko O.; Laskavyi V.; Dykman L. Immunostimulatory effect of gold nanoparticles conjugated with transmissible gastroenteritis virus. Bull. Exp. Biol. Med. 2011, 151 (4), 436 10.1007/s10517-011-1350-8. 22448360 PMC7087599

[ref243] Kim Y.-S.; Son A.; Kim J.; Kwon S. B.; Kim M. H.; Kim P.; Kim J.; Byun Y. H.; Sung J.; Lee J.; et al. Chaperna-mediated assembly of ferritin-based Middle East respiratory syndrome-coronavirus nanoparticles. Front. Immunol. 2018, 9, 1093 10.3389/fimmu.2018.01093. 29868035 PMC5966535

[ref244] Jung S.-Y.; Kang K. W.; Lee E.-Y.; Seo D.-W.; Kim H.-L.; Kim H.; Kwon T.; Park H.-L.; Kim H.; Lee S.-M.; Nam J.-H. Heterologous prime–boost vaccination with adenoviral vector and protein nanoparticles induces both Th1 and Th2 responses against Middle East Respiratory syndrome coronavirus. Vaccine 2018, 36 (24), 3468–3476. 10.1016/j.vaccine.2018.04.082. 29739720 PMC7115429

[ref245] Lin L. C. W.; Huang C. Y.; Yao B. Y.; Lin J. C.; Agrawal A.; Algaissi A.; Peng B. H.; Liu Y. H.; Huang P. H.; Juang R. H.; et al. Viromimetic STING agonist-loaded hollow polymeric nanoparticles for safe and effective vaccination against Middle East respiratory syndrome coronavirus. Adv. Funct. Mater. 2019, 29 (28), 1807616 10.1002/adfm.201807616. 32313544 PMC7161765

[ref246] Wang J. Fast identification of possible drug treatment of coronavirus disease-19 (COVID-19) through computational drug repurposing study. J. Chem. Inf. Model. 2020, 60 (6), 3277–3286. 10.1021/acs.jcim.0c00179. 32315171 PMC7197972

[ref247] Ostaszewski M.; Mazein A.; Gillespie M. E.; Kuperstein I.; Niarakis A.; Hermjakob H.; Pico A. R.; Willighagen E. L.; Evelo C. T.; Hasenauer J.; et al. COVID-19 Disease Map, building a computational repository of SARS-CoV-2 virus-host interaction mechanisms. Sci. Data 2020, 7 (1), 1–4. 10.1038/s41597-020-0477-8. 32371892 PMC7200764

[ref248] Elmezayen A. D.; Al-Obaidi A.; Şahin A. T.; Yelekçi K. Drug repurposing for coronavirus (COVID-19): in silico screening of known drugs against coronavirus 3CL hydrolase and protease enzymes. J. Biomol. Struct. Dyn. 2020, 39 (8), 2980–2992. 10.1080/07391102.2020.1758791. 32306862 PMC7189413

[ref249] Gupta M. K.; Vemula S.; Donde R.; Gouda G.; Behera L.; Vadde R. In-silico approaches to detect inhibitors of the human severe acute respiratory syndrome coronavirus envelope protein ion channel. J. Biomol. Struct. Dyn. 2021, 39, 2617 10.1080/07391102.2020.1751300. 32238078 PMC7171389

[ref250] Ortega J. T.; Serrano M. L.; Pujol F. H.; Rangel H. R. Role of changes in SARS-CoV-2 spike protein in the interaction with the human ACE2 receptor: An in silico analysis. EXCLI journal 2020, 19, 410 10.17179/excli2020-1167. 32210742 PMC7081066

[ref251] Zhang H.; Saravanan K. M.; Yang Y.; Hossain M. T.; Li J.; Ren X.; Pan Y.; Wei Y. Deep learning based drug screening for novel coronavirus 2019-nCov. Interdiscip. Sci.: Comput. Life Sci. 2020, 12, 368–376. 10.1007/s12539-020-00376-6. PMC7266118 32488835

[ref252] Zhou Y.; Hou Y.; Shen J.; Huang Y.; Martin W.; Cheng F. Network-based drug repurposing for novel coronavirus 2019-nCoV/SARS-CoV-2. Cell discovery 2020, 6 (1), 1–18. 10.1038/s41421-020-0153-3. 32194980 PMC7073332

[ref253] Ross K. A.; Brenza T. M.; Binnebose A. M.; Phanse Y.; Kanthasamy A. G.; Gendelman H. E.; Salem A. K.; Bartholomay L. C.; Bellaire B. H.; Narasimhan B. Nano-enabled delivery of diverse payloads across complex biological barriers. J. Controlled Release 2015, 219, 548–559. 10.1016/j.jconrel.2015.08.039. PMC4656048 26315817

[ref254] Lembo D.; Cavalli R. Nanoparticulate delivery systems for antiviral drugs. Antiviral Chem. Chemother. 2010, 21 (2), 53–70. 10.3851/IMP1684. 21107015

[ref255] Odiba A.; Ottah V.; Ottah C.; Anunobi O.; Ukegbu C.; Edeke A.; Uroko R.; Omeje K. Therapeutic nanomedicine surmounts the limitations of pharmacotherapy. Open Medicine 2017, 12 (1), 271–287. 10.1515/med-2017-0041.

[ref256] Parboosing R.; Maguire G. E.; Govender P.; Kruger H. G. Nanotechnology and the treatment of HIV infection. Viruses 2012, 4 (4), 488–520. 10.3390/v4040488. 22590683 PMC3347320

[ref257] Li D.; Johanson G.; Emond C.; Carlander U.; Philbert M.; Jolliet O. Physiologically based pharmacokinetic modeling of polyethylene glycol-coated polyacrylamide nanoparticles in rats. Nanotoxicology 2014, 8 (sup1), 128–137. 10.3109/17435390.2013.863406. 24392664

[ref258] Sanvicens N.; Marco M. P. Multifunctional nanoparticles–properties and prospects for their use in human medicine. Trends Biotechnol. 2008, 26 (8), 425–433. 10.1016/j.tibtech.2008.04.005. 18514941

[ref259] Müller R. H.; Gohla S.; Keck C. M. State of the art of nanocrystals–special features, production, nanotoxicology aspects and intracellular delivery. Eur. J. Pharm. Biopharm. 2011, 78 (1), 1–9. 10.1016/j.ejpb.2011.01.007. 21266197

[ref260] Harris J. M. Poly(Ethylene Glycol) Chemistry: Biotechnical and Biomedical Applications; Springer Science & Business Media, 2013.

[ref261] Han J.; Zhao D.; Li D.; Wang X.; Jin Z.; Zhao K. Polymer-based nanomaterials and applications for vaccines and drugs. Polymers 2018, 10 (1), 31 10.3390/polym10010031. PMC6415012 30966075

[ref262] Schröder M.; Bowie A. An Arms Race: Innate Antiviral Responses and Counteracting Viral Strategies. Biochem. Soc. Trans. 2007, 35 (6), 1512–1514. 10.1042/BST0351512. 18031256

[ref263] Rizvi S. A.; Saleh A. M. Applications of nanoparticle systems in drug delivery technology. Saudi Pharm. J. 2018, 26 (1), 64–70. 10.1016/j.jsps.2017.10.012. 29379334 PMC5783816

[ref264] Carrouel F.; Conte M. P.; Fisher J.; Gonçalves L. S.; Dussart C.; Llodra J. C.; Bourgeois D. COVID-19: A recommendation to examine the effect of mouthrinses with β-cyclodextrin combined with citrox in preventing infection and progression. J. Clin. Med. 2020, 9 (4), 1126 10.3390/jcm9041126. PMC7230644 32326426

[ref265] Zhu M.; Wang R.; Nie G. Applications of nanomaterials as vaccine adjuvants. Hum. Vaccines Immunother. 2014, 10 (9), 2761–2774. 10.4161/hv.29589. PMC4977448 25483497

[ref266] Mishra S. K.; Tripathi T. One year update on the COVID-19 pandemic: Where are we now. Acta Trop. 2021, 214, 105778 10.1016/j.actatropica.2020.105778. 33253656 PMC7695590

[ref267] Singh R.; Kang A.; Luo X.; Jeyanathan M.; Gillgrass A.; Afkhami S.; Xing Z. COVID-19: Current knowledge in clinical features, immunological responses, and vaccine development. FASEB J. 2021, 35 (3), e21409 10.1096/fj.202002662R. 33577115 PMC7898934

[ref268] Villoutreix B. O.; Calvez V.; Marcelin A.-G.; Khatib A.-M. In Silico Investigation of the New UK (B. 1.1. 7) and South African (501Y. V2) SARS-CoV-2 Variants with a Focus at the ACE2–Spike RBD Interface. Int. J. Mol. Sci. 2021, 22 (4), 1695 10.3390/ijms22041695. 33567580 PMC7915722

